# Complex Materials with Stochastic Structural Patterns: Spiky Colloids with Enhanced Charge Storage Capacity

**DOI:** 10.1002/advs.202305085

**Published:** 2023-11-30

**Authors:** Yuan Cao, Bingcheng Luo, Atif Javaid, Hong Ju Jung, Tao Ma, Chung‐Man Lim, Ahmet Emre, Xiaohui Wang, Nicholas A. Kotov

**Affiliations:** ^1^ Department of Chemical Engineering University of Michigan Ann Arbor MI 48109 USA; ^2^ Biointerface Institute University of Michigan Ann Arbor MI 48109 USA; ^3^ College of Science China Agriculture University Beijing 100083 China; ^4^ Department of Polymer Engineering University of Engineering and Technology G. T. Road Lahore 54890 Pakistan; ^5^ Department of Materials Science and Engineering University of Michigan Ann Arbor MI 48109 USA; ^6^ Center for Complex Particle Systems (COMPASS) University of Michigan Ann Arbor MI 48109 USA; ^7^ Michigan Center for Materials Characterization University of Michigan Ann Arbor MI 48109 USA; ^8^ School of Materials Science and Engineering Tsinghua University Beijing 100084 China; ^9^ Department of Macromolecular Science and Engineering University of Michigan Ann Arbor MI 48109 USA; ^10^ Department of Aeronautics Faculty of Engineering Imperial College London South Kensington Campus London SW7 2AZ UK

**Keywords:** Complex particles, biomimetic nanostructures, metamaterials, Structural supercapacitors, Topological reconfiguration

## Abstract

Self‐assembled materials with complex nanoscale and mesoscale architecture attract considerable attention in energy and sustainability technologies. Their high performance can be attributed to high surface area, quantum effects, and hierarchical organization. Delineation of these contributions is, however, difficult because complex materials display stochastic structural patterns combining both order and disorder, which is difficult to be consistently reproduced yet being important for materials' functionality. Their compositional variability make systematic studies even harder. Here, a model system of FeSe_2_ “hedgehog” particles (HPs) was selected  to gain insight into the mechanisms of charge storage n complex nanostructured materials common for batteries and supercapacitors. Specifically, HPs represent self‐assembled biomimetic nanomaterials with a medium level of complexity; they display an organizational pattern of spiky colloids with considerable disorder yet non‐random; this patternt is consistently reproduced from particle to particle. . It was found that HPs can accommodate ≈70× greater charge density than spheroidal nano‐ and microparticles. Besides expanded surface area, the enhanced charge storage capacity was enabled by improved hole transport and reversible atomic conformations of FeSe_2_ layers in the blade‐like spikes associated with the rotatory motion of the Se atoms around Fe center. The dispersibility of HPs also enables their easy integration into energy storage devices. HPs quadruple stored electrochemical energy and double the storage modulus of structural supercapacitors.

## Graphical abstract

‘Hedgehog’ particles from FeSe_2_ with medium level of complexity and functional disorder reveal reversible charge–discharge cycles and 70 times higher charge storage capacity than simple particles that can be used in high‐performance energy storage.

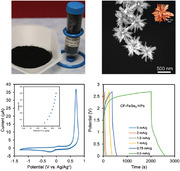

## 1 Introduction

Colloids and surfaces with complex architectures based on spikes, struts, scrolls, platelets, etc. are empirically known to provide performance advantages for supercapacitors,^[^ ^1^, ^2^, ^3^ ^]^ batteries, electrocatalysts, and biosensors compared to near‐spherical particles.^[^ ^4^, ^5^, ^6^, ^7^, ^8^, ^9^ ^]^ The rationale for the advantages of these complex porous architectures with hierarchical organization and nonrandom disorder remains, however, convoluted. Intuitive and theoretical^[^ ^10^ ^]^ arguments can be made that nanostructured and mesostructured materials (see Section S1.1, Supporting Information) have the advantage of high surface area, especially for technologies related to charge storage and redox activity. They also display strong quantum confinement effects due to nanoscale dimensions that can also benefit these functionalities. However, particle size, physical properties of the components, and long‐range and short‐range organization of the materials collectively contribute to materials' performance. There may also be additional factors that can further enhance the performance that could be overlooked. Evaluation of electrochemical properties of materials with complex hierarchical architecture, verification of the basic intuitive hypotheses, and identification of additional physical phenomena are complicated by the multitude of geometrical patterns, synthetic protocols, and materials platforms.^[^ ^3^, ^11^ ^]^ The difficulties in systematic description of their structure and thus the dependences between structure and performance, are reflected by the staggering diversity of terms being used for the particles and surfaces with spikes, struts, and nanosheets. The complex hierarchically organized nano‐, meso‐, micro‐, and macrostructured materials^[^ ^12^ ^]^ with semi‐random but visually identifiable structural patterns are often depicted as flower‐, sponge‐, hedgehog‐, nest‐, honeycomb‐, and spider‐like nanostructures.^[^ ^3^, ^11^, ^12^, ^13^, ^14^ ^]^ Biomimetic parallels in these materials are justifiable and not accidental because the majority of biological materials are taking advantage of nonrandom functional disorders, too. Nevertheless, the comparison of surface charge density and redox potential of, for instance, flower‐like to spider‐like battery anodes or cathodes is non‐trivial and systematic studies in the domain of complex and bioinspired materials with stochastic yet consistently reproduceable structural patterns markedly different than chemists, physisists, and materials scientists are used to encounter (i.e. crystals and glasses), will require a new toolbox for the description of their organization and structure‐property relations.

The difficulty with explaining how organizational complexity affects electrochemical characteristics can be highlighted by the examples of contradictive trends and vagueness of their structural descriptions. For example, supercapacitors made from Ni@NiO hedgehog‐like, nano prickly, and spheroidal particles ^[^ ^15^ ^]^ displayed a performance that contradicts the surface area argument. Similar contradictions can be traced for sponge‐like and flower‐like CuCo_2_O_4_@CuCo_2_S_4_ particles.^[^ ^16^ ^]^ The relationships between the density of spikes, their diameter, length, and connectivity of nanostructured elements versus redox activity and charge accumulation in these examples remain mechanistically intractable even when the quantum size and surface area effects are considered. Analysis of these and other data cited above in respect to the size‐dependent energy gap or the total surface area fail to show consistent electrochemical dependences

A part of the problem is that multiple experimental observations indicate that redox‐active nanostructured particles ^[^ ^17^, ^18^, ^19^, ^20^, ^21^, ^22^, ^23^, ^24^ ^]^ and materials^[^ ^25^, ^26^ ^]^ with stochastic structural patterns may also be chemically unstable upon charging. It makes establising the structure‐property relations even more convoluted; it also highlights very vividly the need to understand the reasons for high performance. For example, the architectures from spikes, struts, and nanosheets can be viewed as fundamentally less suitable for charge storage and redox‐active surfaces than typical compact composites due to the thermodynamic metastability of complex materials.^[^ ^34^ ^]^ Indeed, the metastability of electroactive nanostructures with stochastic structural patterns was observed during the electrochemical cycling of gold hedgehogs,^[^ ^27^ ^]^ Au@MnO_2_ nanowires with a network of “petals”,^[^ ^28^ ^]^ flower‐like Co_x_Mn_y_Ni_z_O_4_^[^ ^29^ ^]^ and other cases.^[^ ^30^, ^31^, ^32^, ^33^ ^]^

Spiky “hedgehog” particles (HPs) from FeSe_2_ provide a convenient model system to address these questions. On one hand, HPs represent high‐performance materials but a medium level of complexity. Their structure can be described using Graph Theory (GT) using the prnciples similar to those used in chemical formulaes, i.e. atomic graphs. GT also enables one to quantify the complexity of their architechture to standardize it among many other possible architechtures with stochastic yet non‐random structural patterns. The complexity index (*CI*) of HPs from FeSe_2_ is 23, which is smaller than for many other particles.^[^ ^34^, ^35^ ^]^ Besides the quantifiable and consistent organization, HPs are easy to make due to strong self‐assembly restrictions based on electrostatic repultion and lattice‐to‐lattice self‐assembly preferences.^[^ ^17^ ^]^ Furthermore, FeSe_2_ HPs retain their geometry both in dispersions and composites displaying high redox activity enabling the evaluation of their electrochemical characteristics, such as charge storage capacity. We find that that high surface area alone cannot explain the 70× increase in charge storage capacity compared to simple spherical particles. There is an additional large component related to the reconfiguration of the FeSe_6_ octahedral units that helps to increase the stability of the HPs with an increasing number of holes in the crystal lattice. Doping of the HP surface with iron increases the conductivity of the surface layer of particles, which improves the charge transport and uniformity of the hole distribution over the particle. The particle dispersibility enables their integration with various devices such as structural supercapacitors.

## 2 Structure of FeSe_2_ HPs

**Figure 1:**
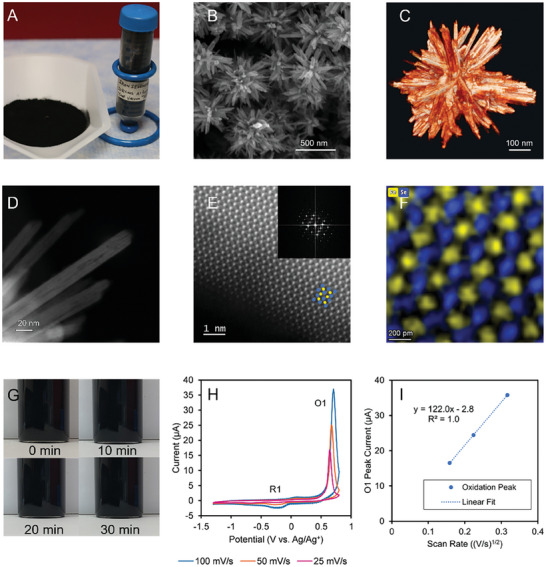
Structure and electrochemical activity of FeSe_2_ HPs. A) Gram‐scale synthesis of FeSe_2_ HPs. B) SEM image of FeSe_2_ HPs. C) Snapshot of tomography reconstruction of a single FeSe_2_ HP. D) HAADF‐STEM image of FeSe_2_ nanosheets forming nano spikes of HPs. E) HAADF‐STEM and F) EDS mapping of FeSe_2_ nanosheet forming a spike of HP showing its single crystalline structure. G) Photographs of 0.2 mg mL^−1^ FeSe_2_ HPs dispersion with 0.1 m LiTf in DMF, in a vial for 0, 10, 20, and 30 min. H) CV of 0.2 mg mL^−1^ FeSe_2_ HPs in DMF adding 0.1 m LiTf, with scan rates of 25, 50, and 100 mV s^−1^, the 5th cycle. I) A plot of O1 peak current (*i_p_*, µA) versus the square root of scan rate (*v^1/2^*, (V/s)^1/2^) in CV tests with different scan rates.

Nanostructured particles with complex but reproducible shapes and uniform dimensions can be synthesized by self‐limited assembly of electrostatically charged nanoparticles.^[^ ^17^ ^]^ Prepared FeSe_2_ HPs (**Figure** **1**A) displayed a diameter of 556 ± 40 nm and high size uniformity with dispersity index as low as 7% (Figure 1B). The geometries and statistical distributions of the spikes on the HPs were consistent between scanning electron microscopy (SEM) and transmission electron microscopy (TEM) data (Figure S1, Supporting Information). Moreover, the 3D geometry assessed by tomography reconstruction (Figure 1C, Video S1, Supporting Information) and 2D TEM modalities (Figure S2, Supporting Information) revealed that these linear crystalline segments spread outward in all directions from the polycrystalline particle core.

The powder diffraction pattern (Figure S3, Supporting Information) closely matches those of theoretical, simulated X‐ray diffraction (XRD) spectra without any additional peaks.^[^ ^36^ ^]^ ICP‐MS analysis (Se 77: 34.350 ppb, Fe 56: 14.650 ppb) indicated that the Fe/Se atomic ratio for HPs is 0.603 confirming previous SEM‐EDS data about small excess iron in the HPs.^[^ ^17^ ^]^ EDS mapping further substantiated the excess of iron in the particles with the Fe/Se atomic ratio of 0.557, with excess Fe atoms present at the edges of the spikes (Figure S4a–c, Supporting Information).

A close examination of the spikes showed that they are blade‐like nanosheets (Figure 1D; Figure S4c, Supporting Information). The high‐resolution HAADF‐STEM image (Figure 1E) and EDS mapping (Figure 1F) confirmed that the spikes are predominantly single crystalline FeSe_2_. A thin amorphous layer was observed at the outer edge of the spike (Figure S4c, Supporting Information) and was confirmed as a Fe‐rich phase (Figure S4b, Supporting Information) by both ICP‐MS and EDS.

Addressing the challenge of particle clumping in high ionic strength media, HPs were easily dispersed in *N, N*‐dimethylformamide (DMF), which is one of the preferred solvents for electrochemical studies. FeSe_2_ HPs retained colloidal stability even in the presence of 0.1 m LiTf that served as the supporting electrolyte (Figure 1G); no additional surfactants or dispersing aids were used. These and other HPs resist irreversible coagulation in hydrophilic, hydrophobic, and high‐ionic‐strength media because stiff spikes reduce van der Waals interactions between the particles.^[^ ^20^, ^21^ ^]^ The stiffness of the spikes in the specific example of FeSe_2_ HPs responsible for higher dispersibility can be vividly appreciated from the electron microscopy images (Figure 1B,D). The morphology of the soft spikes, for instance, from polymers, is strongly affected by the capillary forces during drying,^[^ ^37^ ^]^ which is certainly not the case for HPs from FeSe_2_ or rigid polymers.^[^ ^19^ ^]^ The 3D tomographic reconstructions of HPs with blade‐like spikes are described by the *K_3_* complete graph rather than the *K_2_* complete graph with descriptive or cylindrical spikes.

## 3 Electrochemical Properties of FeSe_2_ HPs

The volumetric content of FeSe_2_ HPs used in all our cyclic voltammetry (CV) experiments was 0.2 mg mL^−1^, which corresponds to a particle concentration of 4.4 × 10^−15^ mol cm^−3^, as established by particle tracking analysis (PTA, Sections S2.1 and S2.2, Supporting Information). The uncompensated resistance (*R_u_*) of the dispersion containing 0.2 mg mL^−1^ FeSe_2_ HPs in DMF with 0.1 m LiTf was determined by electron impedance spectroscopy (EIS) in the frequency range from 700 kHz to 1 Hz (Figure S5, Supporting Information) and was found to be ≈20 Ω, which can be neglected for reliable measurements in liquid electrolytes (Section S3.1, Supporting Information).

In a typical CV scan (illustrated in Figure S6, Supporting Information), the principal oxidation (O1) peak and the minor reduction peak (R1) of FeSe_2_ HPs were observed at 0.68 ± 0.04 V and −0.17 ± 0.02 V (vs Ag/Ag^+^) (Figure 1H). These peaks correspond to electron withdrawal from the conduction band and their injection into the valence band, respectively. The R1–O1 separation of 0.85 ± 0.06 V falls within the range of FeSe_2_ bandgap energies from 0.67 to 0.86 eV.^[^ ^38^ ^]^ No additional peaks were recorded before the first anodic half scan (Figure S7a, Supporting Information), which demonstrates that the R1 peak is associated with the reduction of oxidized FeSe_2_ HPs at O1. CV scans between −1.3 V and 0.2 V (vs Ag/Ag^+^) with the same scan rate of 100 mV s^−1^ (Figure S7b, Supporting Information) also confirm the attribution of the peaks. A linear relationship between the peak current (*i_p_*) versus the square root of scan rate (*v^1/2^*) and the peak shift of O1 to higher energies at faster scans was observed by varying the CV scan rate from 25 mV to 100 mV s^−1^ (Figure 1I). These trends indicate that 1) electron transfer between HPs and electrodes is limited by mass transport in dispersion^[^ ^39^ ^]^ and 2) HPs are not irreversibly adsorbed on the surface of the gold disk working electrode during the redox processes.^[^ ^39^ ^]^

## 4 Structural Changes in HPs During Redox Cycling

**Figure 2:**
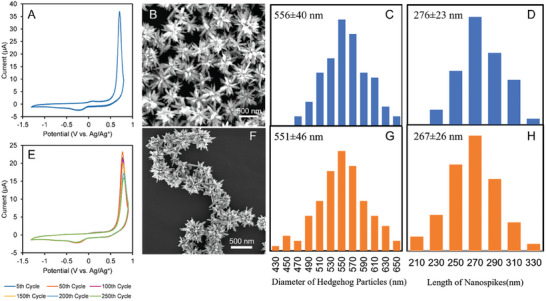
Cyclic voltammetry and morphology of FeSe_2_ HPs. A) CV for the 5th cycle. B) SEM image of HPs after the 5th cycle. Histograms for C) diameter and D) nanospike length distributions for HPs after short‐term CV tests. E) CV for continuous cycling over 250 cycles. F) SEM image of FeSe_2_ HPs after the 250th cycle. Histograms for G) diameter and H) nano spikes length distributions for HPs after long‐term CV tests. For all CV scans in this dataset, 0.2 mg mL^−1^ FeSe_2_ HPs were dispersed in DMF with 0.1 m LiTf, scan rate of 100 mV s^−1^.

The morphology of HPs was analyzed after the end of the 5th cycle (**Figure** **2**A–D) in the series of 250 CV scans. SEM images of HPs after the 250th cycle (Figure 2E) indicated that the nanoscale structures of HPs remain intact (Figure 2F), which is also evidenced by histograms of structural features (Figure 2G,H) for HPs before and after 250 CV scans. The average diameter of HPs was 551 ± 46 nm and the average length of nanospikes was 267 ± 26 nm after the 250‐cycle CV experiments, which is statistically identical to HPs prior to charge‐discharge cycling. The overlapping voltammograms of HPs during the CV scans substantiated the electrochemical stability of FeSe_2_ HPs within a wide range of potentials that were problematic for other mesoscale particles and surfaces.^[^ ^27^, ^28^, ^29^ ^]^

## 5 Charging Capacity of FeSe_2_ HPs

**Figure 3:**
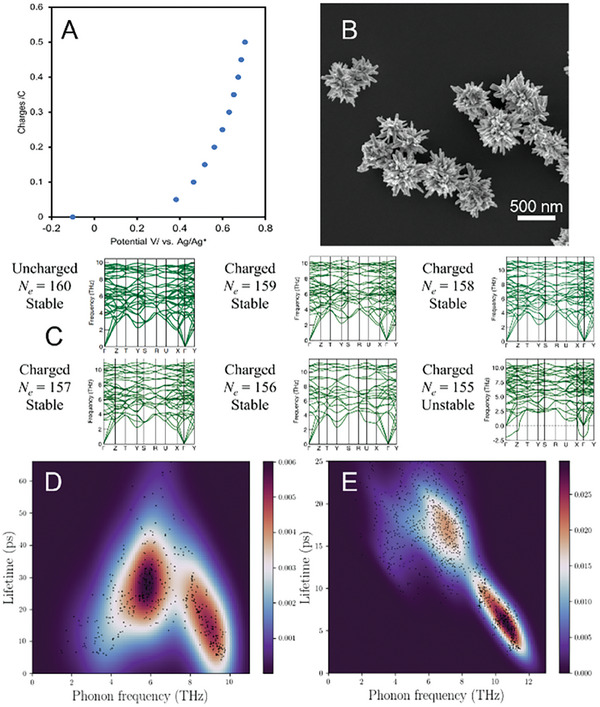
Charging capacity of FeSe_2_ HPs. A) Bulk electrolysis plot of 0.3 mg mL^−1^ FeSe_2_ HPs and B) their SEM image after in DMF. C) Phonon dispersion relations of FeSe_2_ supercell with different amounts of excess charges. When *N_e_* = 155, the supercell becomes unstable. Phonon lifetime of D) uncharged (*N_e_* = 160) and E) charged (*N_e_* = 155) FeSe_2_ HPs at 300 K.

The number of charges that can be stored on a particle without its chemical or physical disintegration is an essential attribute of electroactive nanoscale and microscale colloids. We calculated the number of charges stored on HPs based on the bulk electrolysis experiments recording open circuit potential by gradually increasing the amount of charge passed onto the particles. We found that the maximum number of positive charges transferred onto an HP without any loss of its physical integrity is as high as 1.5 × 10^8^ (**Figure** **3**A,B, and Section S4.1, Supporting Information), which demonstrates the impact of complex interface‐rich particle architecture for its charge storage capacity.

There is no prior data on the charging capacity of HPs or similarly complex colloidal particles . One benchmark would be to compare them with the charge storage capacity data of semiconductor nanoparticles known as quantum dots (QDs). The number of charges stored on HPs is ≈10^6^ times greater than on QDs.^[^ ^40^, ^41^ ^]^ HPs are, of course, larger and have ≈4.4 × 10^4^ greater number of crystalline units.^[^ ^40^ ^]^ It will be important, then, to compare the number of charges accommodated in the particle per surface area and per volume. By using the SEM and TEM images of FeSe_2_ HPs, we were able to find that the average length of nanospikes is 276 nm and the mean diameter of nanospikes is 21 nm. (Figure S8, Supporting Information) A FeSe_2_ HP made from FeSe_2_ nanosheets, carrying an average of 200 nano spikes, has a total volume (including the core and the spikes) of 1.2 × 10^7^ nm^3^ (Section S4.2, Supporting Information), which corresponds to an average of 2.2 × 10^8^ FeSe_2_ unit cells. Therefore, the number of charges per volume in the highest charged state corresponds to ≈13 charges nm^−3^ (Table S1, Supporting Information), while this parameter for QDs is from 1 to 1.5 charges nm^−3^.^[^ ^40^, ^41^ ^]^ Similar trends were noticed for charges per surface area of a single particle (Table S2, Supporting Information).

Based on the data for CdS QDs reported by Haram et al.,^[^ ^40^ ^]^ the number of electrons stored per CdS unit cell is ≈0.01 (Section S4.5, Supporting Information). In contrast, the number of charges stored per unit cell for FeSe_2_ HPs is ≈70 times higher. The comparison with QD indicates that the quantum size effect per se does not make a large contribution to the increase of charge storage capacity, but they do change the redox potential substantially.

It is also instructive to compare the charging capacity of HPs with that of metal colloids of similar size (Section S4.6, Supporting Information). For example, a gold particle with a diameter of 1 µm dispersed in water can store only 10^6^ charges, which is ≈100 times fewer than that for HPs. These experimental data and simple calculations (see SI) clearly indicate that surface area alone cannot explain the high charge storing capacity and, more generally, the advantages of complex materials with stochastic structural patterns for energy technologies.

Chemical structure of highly charged states in FeSe_2_ HPs. HPs retain both physical integrity and colloidal stability in their highly charged state. Besides high surface area and colloidal stability, the redox chemistry of FeSe_2_ HPs must also be significant for high charge storage capacity. We carried out density functional theory (DFT) and density functional perturbation theory (DFPT) calculations^[^ ^42^, ^43^ ^]^ based on the structural data about the FeSe_2_, to assess the stability of the atomic structure of FeSe_2_ HPs with different excess positive charges. Their accuracy was benchmarked by the comparison of experimental^[^ ^17^ ^]^ and computational data. For example, we compared calculations of XRD patterns and found good agreement between theoretical and experimental data (Figure S3, Supporting Information).

The total number of valence electrons (*N_e_*) for uncharged FeSe_2_ with no excess electrons was 160 per 2×2×1 supercell (Figure 3C, Supporting Information). From X‐ray photoelectron spectroscopy (XPS), we infer that bulk electrolysis results primarily in the oxidation of Se atoms, while the redox state of Fe atoms remains largerly unchanged (Figures S11 and S12, Supporting Information). To model the oxidation of Se atoms in FeSe_2_ nanosheets forming HPs , we gradually reduced the number of valence electrons in the supercell (Figure S11, Supporting Information) and calculated phonon dispersion along the high‐symmetry lines in the Brillouin zone.^[^ ^44^ ^]^ The transition of vibrational frequencies of phonons from simple positive values to imaginary phonon frequencies marks the threshold of the chemically stable atomic lattice.^[^ ^42^, ^45^, ^46^, ^47^ ^]^ We found that the atomic structure of FeSe_2_ remained stable until the positive charge exceeded four electrons per the model (*N*_e_ < 156). When *N_e_* in the FeSe_2_ supercell was reduced to 155, the imaginary phonon frequencies appeared (Figure 3C). The phonon lifetime of FeSe_2_ calculated using first‐principles anharmonic lattice dynamics calculations (Figure 3D,E) strongly decreased upon charging, which indicated that the lattice thermal conductivity essential for many catalytic reactions is concomitantly reduced (Figure S12, Supporting Information).^[^ ^48^ ^]^ Note that the stability limit for FeSe_2_ obtained in the computations corresponds to 0.5 charges per unit cell, which is quite similar to the experimentally determined excess charge of 0.7 charges per FeSe_2_.

**Figure 4:**
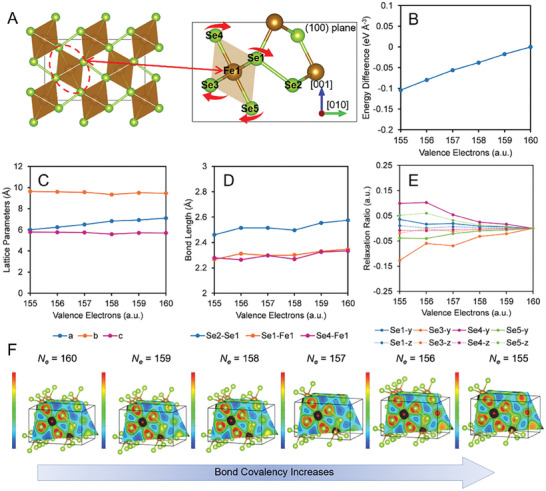
Chemical structure changes at a highly charged state on FeSe_2_ HPs. A) The geometry lattice model of charged FeSe_2_ supercell. The red arrows show the rotation under the effects of excess charges. B) The total energy difference, C) lattice parameters, D) bond lengths, and E) ionic relaxation ratios at the dependence of excess charges on FeSe_2_ supercell. F) Electron charge density mapping with increasing excess charges.

FeSe_2_ HPs have a space group of Pnnm and a point group of D2h (mmm) (**Figure** **4**A, Video S2, Supporting Information) with two Wyckoff positions: 2b site for Fe atom and 4 g site for Se atom. The DFT/DFPT computations for highly charged states *N*_e_ = 156 showed that [FeSe_6_] octahedrons rotated clockwise (Figure 4A, Video S3, Supporting Information) in the (100) plane as excess positive charges increased. The total energy difference decreased as *N*_e_ decreased, but the main lattice parameters remained nearly constant throughout the charging process (Figure 4B,C), which is consistent with experimental observations of the physical integrity of an HP in a highly charged state. Various Fe‐Se and Se‐Se bond lengths also change little upon charging (Figure 4D). Large displacements of electron density were observed, however, around Se sites along the [010] and [001] directions (Figure 4E), and no changes occurred along the [100] direction. Similarly, the changes in the Se ionic relaxation ratio along the [010] direction were larger than that along the [001] direction. At the same time, we also observe the distinct rotatory motion of the Se atoms around the Fe center as excess charges increase, evidenced by the ion relaxation ratio (Figure 4E, Table S3, Supporting Information). Such rotation is facilitated by the specific topology of the lattice with low dimensionality of the FeSe_2_ in the spikes with dominant 2D nanoscale geometry (Figure 1C). Note that identification of this mechanism would not be possible in other nanostructured materials with stochastic structural patterns, because of the huge variability of materials organization.

Evaluating the partial density of states, we also established a large change in the hybridization between the Fe 3d states and Se 3p states. The overlap between conduction and valence bands increased with an excess of positive charge pointing to higher conductivity of HPs in highly charged states (Figure S13, Supporting Information). The states of Fe and Se at the conduction band minimum concomitantly shifted to lower energy (Figure S14, Supporting Information). With that, the covalent character of the Se‐Se bonds increased as can be seen in the charge density mappings (Figure 4F), which also enhanced the chemical stability of the charged HPs. The excess iron atoms observed from the chemical composition help to increase the chemical stability, which contributes to the high charge storage capacity because it facilitates uniform distribution of charges over the entire HP.

We note that the reconfiguration of the lattice of that [FeSe_6_] octahedrons is identical to those in macroscale topological metamaterials enabling the latter to accommodate large stresses.^[^ ^49^, ^50^, ^51^, ^52^ ^]^ The large surface area, typical for complex networked materials with nanostructured struts, fibers, and spikes, simplifies such atomic reconfiguration due to the smaller energy of reconfiguration for atoms at interfaces than in the bulk.

**Figure 5:**
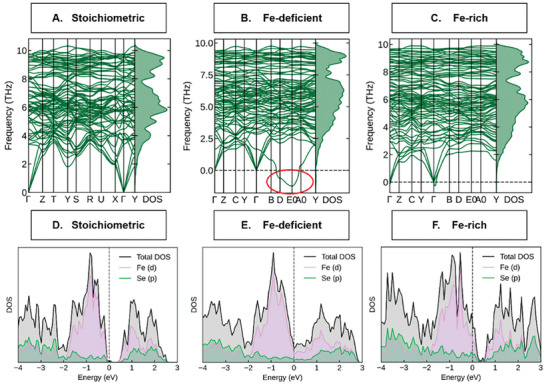
(A,B,C) The phonon dispersion and phonon density of states of FeSe_2_ A), Fe_0.875_Se_2_ B), and Fe_1.067_Se_2_ C). The red oval highlights the area with negative frequencies in B. (D,E,F) Partial density of states (DOS) of FeSe_2_ D), Fe_0.875_Se_2_ E) and Fe_1.067_Se_2_ F).

The DFT/DFPT computations also afford examination of the role of iron doping in FeSe_2_ HPs observed in XPS (Figures S9 and S10, Supporting Information) and SEM‐EDS data (Figure S4, Supporting Information). The analysis of phonon dispersion and phonon density of states demonstrates that iron selenide with excess of Fe maintains stability with minimal imaginary frequencies, while structure deficient in of Fe is unstable along the B‐D‐E0‐A0 *k*‐points in the first Brillouin with large imaginary frequencies (**Figure** **5**). It is also observed that the lattice structure maintains stability at the Γ point for both structures, this means no matter how small contents of Fe ions are added to or reduced from the FeSe_2_, little effects were done on the lattice structure stability at Γ point.

HPs are reversibly oxidized in the charging process and, therefore, the hole conductance is most significant in this case.  The density of states (DOS) forming the valence band in Fe‐rich HPs a) increases compared to stoichiometric particles and b) crosses the Fermi level set at zero (Figure 5).  Both electronic effects lead to an increase of hole conductance with Fe doping, which was also observed in previous studies.^[^ ^53^, ^54^ ^]^

The charge carrier states in both valence and conduction bands in the vicinity of the band gap originate from Fe atoms, which indicates that charge transport in HPs and other forms of iron diselenide occurs predominantly through the planes formed by Fe atoms on the orthorhombic cell of FeSe_2_. The calculations agree with experimental observations of multiple charge‐discharge cycles, which are enabled by the well‐known reversibility of redox processes involving Fe^3+^.

The fast transport of holes in the surface layer of HPs greatly facilitates their charging process. It enables holes to be rapidly and uniformly distributed over their complex topographic features. The increased conductivity related to Fe doping is a kinetic factor that accelerates and benefits the charging but does not determine the ultimate hole density and charge storage capacity of HPs. The latter is determined by the structural and chemical stability of HPs, especially in a cyclic charge‐discharge process.

## 6 HPs in Structural Supercapacitors

The combination of the large charge storage capacity of HPs prompts their utilization in energy storage devices. Multifunctional batteries and supercapacitors that can store high amounts of charge and carry a large tensile load simultaneously which are known as structural supercapacitors and structural batteries are some of the most challenging ones. These energy storage devices are quintessentially biomimetic replicating multifunctional energy storage in living organisms.^[55]^ Enabling net weight savings compared to unifunctional energy storage units, these devices are needed for drones and other robots to extend their operational time.

Considering structural energy storage, FeSe_2_ HPs have three advantages over other redox materials: First, they not only have high charge storage capacity but can also be made in vast quantities due to the self‐restricted mode of their self‐assembly.^[^ ^17^ ^]^ Second, the spiky topology of HPs facilitates connectivity between particles and, thus, the charge transport within the active layer. Third, the rigid crystalline spikes promote connectivity of HPs with epoxy resin serving as a solid electrolyte in these devices. Prior studies consistently show improvements in the mechanical properties of gels and composites from particles with spiky and non‐spherical shapes.^[^ ^56^, ^57^, ^58^, ^59^, ^60^ ^]^

**Figure 6:**
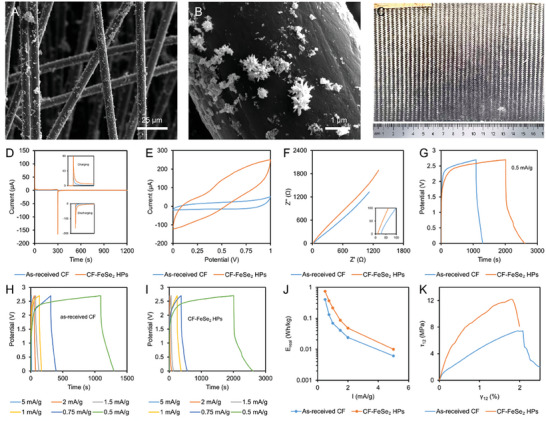
FeSe_2_ HP films deposited on carbon fabric (CF) and the electrochemical and mechanical properties of HP‐based structural supercapacitors. SEM images of FeSe_2_ HPs deposited onto carbon fabrics at a magnification of A) 350× and B) 25 000×. (Also see the X‐ray tomography reconstructions in Videos S4 and S5, Supporting Information. C) Photograph of a structural supercapacitor on carbon fabric in A4 format. D) Chronoamperometric charge‐discharge test, E) CV test at a scan rate of 100 mV s^−1^, F) EIS test, G) Galvanostatic charge‐discharge (GCD) test at a current density of 0.5 mA g^−1^. GCD test at different current densities for structural supercapacitor with H) as‐received CF electrodes and I) CF‐FeSe_2_ HPs electrodes. J) Real specific energy at different current densities, and K) in‐plane shear stress as a function of the in‐plane shear strain of the fabricated structural supercapacitors.

FeSe_2_ HPs were deposited onto two pieces of carbon fabric (CF, **Figure** **6**A–C) that were subsequently bonded with the ion‐conductive epoxy resin in between. The resulting structural supercapacitors were tested with respect to charge storage and load‐bearing performance. X‐ray tomography video (Video S4, Supporting Information) demonstrated that FeSe_2_ HPs were successfully integrated into the device. We also note that the distribution of the HPs in the fabric was not conducive to high‐performance energy storage due to sub‐optimal processing conditions, yet the charge storage tests were informative, promising and highly competitive with previously reported devices of similar type.

Despite the low overall loading of HPs on the carbon fabric and distinct clustering, the chronoamperometry charge‐discharge and cyclic voltammetry tests (Figure 6D,E) indicate that FeSe_2_ HPs substantially increase the amount of the stored charge and energy density of the supercapacitor. The gravimetric and volumetric specific capacitances of the fabricated devices were improved by 3.3 and 3.6 times, respectively, compared to CF supercapacitor without active particles. In accordance with the theoretical expectations,^[^ ^61^, ^62^ ^]^ the Nyquist plot (Figure 6F), and galvanostatic charge‐discharge plots (Figure 6G–I) confirm that the charge transport kinetic is facilitated after the incorporation of FeSe_2_ HPs. The specific energy of the devices increased with the decreasing current densities (Figure 6J). The device performance is highly competitive compared to the specific energy and power of other structural supercapacitors (Figure S15 and Table S4, Supporting Information).

Note that the energy storage and membrane‐based devices often reveal a scaling problem: performance parameters for small‐ and large‐scale cells can differ dramatically due to low‐probability but high‐impact defects (i.e. stochastic holes) in the membranes.^[^ ^65^ ^]^ Thus, we decided to test the performance of FeSe_2_ HPs in both types of supercapacitors. Fully consistent with the electrochemical data above, the small‐scale Swagelok cells and the large‐scale tablet‐like cells in A4 format (Figure S16, Supporting Information) show distinct improvements in the device‐level performance compared to the CF‐only devices when HPs are incorporated.

Another parameter space needed for the multifunctionality of the energy storage devices is related to mechanical properties. The increase of modulus or strength of the electroactive matrix in structural supercapacitors is typically achieved at the expense of the charge transport capabilities^[^ ^63^ ^]^ and the utilization of the spiky particles enables one to obviate this property dilemma.^[^ ^64^ ^]^ The complex spiky morphology of the HPs also leads to the concomitant increase in the in‐plane shear modulus (Figure 6K) and impact strength (**Table** **1**).

**Table 1:** Effect of FeSe_2_ HPs loading on the electrochemical properties, including specific capacitance *C_V_*, gravimetric capacitance *C_g_*, real specific energy *E_real_*, equivalent series resistance *R_s_*, and power density *P*. The mechanical properties, including impact strength *IS*, in‐plane shear modulus *G_12_*, in‐plane shear strength at 0.5% shear strain $\tau_{12}^{0.5\%}$, maximum in‐plane shear strength $\tau_{12}^{m}$ of the structural supercapacitors.

Samples	FeSe_2_	*C_v_* ^a)^	*C_g_* ^a)^	*E_real_*		*R_s_*	*P*		*IS*	*G*_12_	$\tau_{12}^{0.5\%}$	$\tau_{12}^{m}$
	mg cm^−2^ of CF	mF cm^−3^	mF g^−1^	mWh L^−1^	mWh kg^−1^	Ω	kW L^−1^	kW kg^−1^	kJ m^−2^	MPa	MPa	MPa
Carbon Fiber (CF)	0	30.1	23.2	515.8	398.6	25.4 ± 0.1	1.6	1.2	123.2 ± 1.6	1286.3 ± 35.4	3.1 ± 0.3	7.2 ± 0.5
CF + FeSe_2_ HPs	0.2	107.1	77.6	1027.8	744.7	10.1 ± 0.1	1.8	1.3	329.6 ± 3.1	1849.1 ± 79.9	6.1 ± 0.6	11.3 ± 0.7

^a)^ *C_v_*, and *C_g_* are based on the mass and volume of the device respectively, and are reported from the chronoamperometry test; *R_s_* and *P* are reported from EIS; and *E_real_* is reported from the GCD test (at a current density of 0.5 mA g^−1^).

The long‐term cyclic test (Figures S17 and S18, Supporting Information) of structural supercapacitors with FeSe_2_ HPs revealed high Coulombic efficiencies of >97.3% at 1 mA g^−1^, which is promising when compared with similar devices (Table S5, Supporting Information). Capacity retention of 36.4% after 10000 GCD cycles is low compared to “soft” thin film supercapacitors^[^ ^31^ ^]^ but is favorable in the context of “hard” structural supercapacitors; some studies report capacity retention above 90% while the starting capacity is one orders of magnitude smaller (Table S5, Supporting Information). The fact that our devices reached 10 000 GCD cycles—the longest cyclability test for structural supercapacitors up to—is also notable, and FeSe_2_ HPs still exist on carbon fiber matrix largely after the 10000 long cycles. (Figure S19, Video S5, Supporting Information).

## 7 Conclusion

The standardization of complex organization of self‐assembled nanostructures with non‐crystalline function‐critical structural patterns  , as exemplified by FeSe_2_ HPs, enabled in‐depth studies of the electrochemical processes occurring in materials combining order and disorder. We found that HPs possess charging capacity exceeding that of nanoscale and microscale colloids calculated per unit of crystalline lattice, as well as per surface area and volume of a single particle. Besides surface area, an order of magnitude increase of charge storage capacity in FeSe_2_ HPs is enabled by the concerted reconfiguration of the [FeSe_6_] octahedrons upon charging directly reproducing reconfigurability of macroscale mechanical metamaterials. The charging kinetics is accelerated by iron doping in the surface layer of the spikes. The concomitant increase in the particle conductivity increases the uniformity of charge distribution with the active layer and reduces the formation of inaccessible zones. The high charge storage capacity of complex particles can be successfully transferred to the devices, such as structural supercapacitors. Further improvements in the device performance can also be expected from uniform large loading of HPs into the carbon fabric. Further quantification of the complex structures with functional stochasticity and/or nonrandom disorder can accelerate the engineering of a wide range of electroactive and other high‐performance materials.

## Conflict of Interest

The authors declare no conflict of interest.

## Author Contributions

N.A.K. conceived the project. Y.C. and N.A.K. designed the experiments. Y.C. did the synthesis of FeSe_2_ HPs, studied their electrochemical properties, completed all types of characterizations, and analyzed the experimental data. A.J. did the bulk synthesis of FeSe_2_ HPs and deposited these synthesized HPs onto the carbon fabric electrodes, fabricated the structural supercapacitors, and studied their electrochemical properties. C.M.L. did the dynamic mechanical testing of structural supercapacitors. H.J.J. conducted an analysis of the morphological and crystalline structure of FeSe_2_ HPs and examined their dispersibility in solvents. T.M. performed STEM and EDS analysis. A.E. did the SEM of FeSe_2_‐deposited carbon fabrics. B.L. performed the DFT/DFPT simulations of the charged and uncharged FeSe_2_ HPs using the package provided by X.W. All authors co‐wrote the original draft paper.

## Supporting information

Supporting Information

Supplemental Video 1

Supplemental Video 2

Supplemental Video 3

Supplemental Video 4

Supplemental Video 5

## Data Availability

The data that support the findings of this study are available from the corresponding author upon reasonable request.
