# Supplementary material for: Complex Materials with Stochastic Structural Patterns: Spiky Colloids with Enhanced Charge Storage Capacity
Source: Adv Sci (Weinh). 2023 Nov 30;11(4):2305085. doi: 10.1002/advs.202305085 (PMC10811480; doi:10.1002/advs.202305085)
Supplement: Supplementary file 1 — Supporting Information [file ADVS-11-2305085-s004.pdf]

ADVANCED SCIENCE
Open Access

Supporting Information

for *Adv. Sci.*, DOI 10.1002/advs.202305085

Complex Materials with Stochastic Structural Patterns: Spiky Colloids with Enhanced Charge Storage Capacity

*Yuan Cao, Bingcheng Luo, Atif Javaid, Hong Ju Jung, Tao Ma, Chung-Man Lim, Ahmet Emre, Xiaohui Wang and Nicholas A. Kotov**

Supporting Information

Complex Materials with Stochastic Structural Patterns: Spiky Colloids with Enhanced Charge Storage Capacity

Yuan Cao[1,2], Bingcheng Luo[3], Atif Javaid[1,2,4,5], Hong Ju Jung[1,2,6], Tao Ma[5,7], Chung-Man Lim[2,5,6], Ahmet Emre[1,2,6], Xiaohui Wang[8], Nicholas A. Kotov[1,2,5,6,9,10*]

[1]Department of Chemical Engineering, University of Michigan, Ann Arbor, 48109, United States;
[2]Biointerface Institute, University of Michigan, Ann Arbor, 48109, United States;
[3]College of Science, China Agriculture University, Beijing, 100083, China;
[4]Department of Polymer Engineering, University of Engineering and Technology, G. T. Road, Lahore, 54890, Pakistan;
[5]Department of Materials Science and Engineering, University of Michigan, Ann Arbor, 48109, United States;
[6]Center for Complex Particle Systems (COMPASS), University of Michigan, Ann Arbor, MI, 48109, United States;
[7]Michigan Center for Materials Characterization, University of Michigan, Ann Arbor, 48109, United States;
[8]School of Materials Science and Engineering, Tsinghua University, Beijing, 100084, China;
[9]Department of Macromolecular Science and Engineering, University of Michigan, Ann Arbor, 48109, United States;
[10]Department of Aeronautics, Faculty of Engineering, Imperial College London, South Kensington Campus, London, SW7 2AZ.

*Corresponding author: kotov@umich.edu (N.A.K.)

Table of Contents

1. Materials and methods 4

1.1 Nomenclature 4

1.2 Chemicals 4

1.3 Synthesis of $FeSe_2$ HPs 5

1.4 Deposition of $FeSe_2$ HPs on carbon fabrics 5

1.5 Fabrication of structural supercapacitors 6

1.6 Characterization 6

1.7 First-principles calculations 11

2. Determination of particle concentration of $FeSe_2$ Hedgehog Particles 13

2.1 Particle tracking analysis (PTA) 13

2.2 Calculation of particle concentration and averaged molar mass of $FeSe_2$ HPs 13

3. Electrochemical properties of $FeSe_2$ HPs in dispersion 15

3.1 Electrochemical Impedance Spectroscopy (EIS) and Nyquist plot of $FeSe_2$ HPs in DMF 15

3.2 Illustration of reaction of $FeSe_2$ HPs in dispersion 17

3.3 Cyclic Voltammograms (CV) of $FeSe_2$ HPs in DMF 18

4. Calculation of charging capacity 19

4.1 Charging capacity of a single $FeSe_2$ HP 19

4.2 Gravimetric estimation of $FeSe_2$ HPs 19

4.3 Comparison of charges per volume of a single particle 21

4.4 Comparison of charges per surface area of a single particle 22

4.5 Comparison of charges per crystal unit cell for $FeSe_2$ HP and quantum dots 23

4.6 Calculation of charge stored for a metal particle 25

5. Structural characterization of the highly charged states on $FeSe_2$ HPs 26

5.1 X-ray Photoelectron Spectroscopy of Fe and Se on $FeSe_2$ HPs upon charging 26

5.2 X-ray diffraction (XRD) of $FeSe_2$ with different amounts of excess charges 27

5.3 Selenium atoms displacement with different amounts of excess charges 28

5.4 Lattice thermal conductivity of $FeSe_2$ upon charging 29

5.5 Density of States (DOS) of Fe 3d orbital & Se 3p orbital with different amounts of excess charges 30

6. Application of $FeSe_2$ HPs in structural supercapacitor 32

6.1 Comparison of structural supercapacitors with the literature 32

6.2 Electrochemical properties of macroscale tested structural supercapacitors 33

6.3 Cyclic stability experiment of CF- $FeSe_2$ HP structural supercapacitor 34

7. Supplementary References 38

1. Materials and methods

1.1 Nomenclature

In the context of this manuscript, materials, particles, and surfaces displaying structural patterns with characteristic features of 1-100 nm and 50-1000 nm will be referred to as nanostructured and mesostructured, respectively. The terminology in different studies in non-unique and ambiguous about the range between 50 and 100 nm.

1.2 Chemicals

Iron (III) chloride (reagent grade, 97%), oleic acid (technical grade, 90%), 1-octadecene (technical grade, 90%), *N*,*N*-dimethylformamide (ACS reagent, ≥99.8%), lithium trifluoromethanesulfonate (LiTf, 99.95%, trace metals basis), chloroform (HPLC plus, ≥99.9%), oleylamine (technical grade, 70%), 1-ethyl-3-methylimidazolium bis(trifluoromethyl sulfonyl)imide (EMITFSI, HPLC, ≥98%), propylene carbonate (PC, HPLC, 99.7%), poly(ethylene glycol) diglycidyl ether (PEGDGE, Mn ~ 500), and triethylenetetramine (TETA, 60%, technical grade) were purchased from Sigma-Aldrich. Celgard trilayer microporous membrane (H1612, 16 µm thickness, 44% porosity) separator was purchased from Celgard, LLC. 1-dodecanethiol (≥98%) was purchased from Aldrich. Selenium powder (200 mesh, 99.999% (metal basis), Alfa Aesar) and lithium bis(trifluoromethyl sulfonyl)imide (≥98%, Alfa Aesar) were purchased from Fisher Scientific. Twill weave carbon fabric (2×2, 3K, 200gsm, polyacrylonitrile precursor, 97% w/w of carbon, <3% w/w of sizing, electrical conductivity of ~650 mS/cm) was purchased from Fibre Glast Developments Corporation. No further purification was needed for materials received.

1.3 Synthesis of $FeSe_2$ HPs

In a typical synthesis reaction, 0.8110 g iron (III) chloride, 1 mL oleic acid, 20 mL 1-dodecanethiol, 40 mL oleylamine, and 60 mL 1-octadecene were added in a 250 mL 3-neck flask in an argon atmosphere. The mixture was stirred slowly, and the temperature of the solution increased from room temperature to 175 °C in around 30 min. During the process, Fe^{3+} was reduced to Fe^{2+}, and the color of the mixture changed from dark to clear brown with increasing temperature, assuring the reduction of Fe^{3+}. At the same time, 0.7900 g selenium powder was mixed with 14 mL oleylamine and 6 mL 1-dodecanethiol, and the solution needed to be mixed well before adding into the 3-neck flask. When the temperature of solution in the 3-neck flask reached 175°C, the selenium solution was added immediately into the 3-neck flask. The color of the solution turned black suddenly, indicating the formation of $FeSe_2$ nanoparticles. The mixture was stirred slowly for 120 min for $FeSe_2$ to grow into HPs. After the synthesis, the 3-neck flask needed to be cooled down to room temperature rapidly. The suspension was washed three times by centrifugation using chloroform as a washing agent. Then, the black suspensions were exchanged for three times by ethanol to remove chloroform. Next, the dispersions were exchanged for at least three times by water to remove ethanol. The final suspensions in water were frozen in the freezer before drying under vacuum for 48 hours. The black precipitates were harvested and stored inside the glove box for future use.

1.4 Deposition of $FeSe_2$ HPs on carbon fabrics

The hydrophilicity of a 40×20 cm as-received carbon fabric was initially improved through soaking in nitric acid (HNO_3, 67%) at 120°C for 120 min. Nitric acid-treated carbon fabric was washed eight times using water to remove the nitric acid residue and was then dried in a vacuum oven at 60°C

for 24 hours. $FeSe_2$ HPs were then deposited on nitric acid-treated carbon fabrics through a hydrothermal method. 8.0 g $FeSe_2$ HPs were dispersed into 100 mL of deionized water and the mixture, along with the acid-treated carbon fabric, was transferred to a 300 mL autoclave with the Teflon lining and was further treated at 200°C for 48 hours. The obtained carbon fabric was finally washed with water and ethanol and was dried at 60°C under vacuum for 24 hours. The carbon fabric was cut into two equal parts and were used as electrodes. A $FeSe_2$ HPs coating density of 0.2 mg/cm^2 of carbon fabric was measured.

1.5 Fabrication of structural supercapacitors

The structural supercapacitors were fabricated through vacuum bagging method as reported previously.[1] Initially, polymer electrolyte was prepared by mixing 1M LiTFSI/PC (1.0 wt%), EMITFSI (1.0 wt%), PEGDGE (89.2 wt%) and TETA (8.8 wt%). The prepared polymer electrolyte was immediately applied on two $FeSe_2$ HPs deposited carbon fabrics, separated by a Celgard membrane separator. The prepared device was then placed inside vacuum bag and was left overnight for crosslinking at room temperature.

1.6 Characterization

The electrochemical properties of $FeSe_2$ HPs were tested with CV, EIS and bulk electrolysis, carried out by Autolab PGSTAT302N combined with a software NOVA (Metrohm Autolab) to set up different procedures, record data and initially analyze the data. CV and EIS tests were conducted by a three-electrode system containing a working electrode (3 mm gold disk, MF-2114, BASi), a counter electrode (platinum coil auxiliary electrode, MW-1033, BASi) and a reference electrode (Ag/Ag^+, MF-2062, BASi). In the bulk electrolysis experiments, the working electrode was held at 1.0 V vs. Ag/Ag^+

(reference electrode, MF-2062, BASi), below the higher limit for the solvent in order to maintain a maximum driving force. The working electrode applied here was a homemade gold-coated reticulated vitreous carbon (RVC) electrode because there were not commercially available highly porous gold electrodes. Therefore, we used the SPI Sputter Coater to coat the RVC electrode (45 Pores Per Inch (PPI), Low Flow, McMaster Carr, previously cut into thin piece) for 120 seconds on each side. After the coating, the as-fabricated working electrode was dried in the atmosphere overnight before use. The bulk electrolysis tests were followed a similar procedure described in a previous study.[2]

Structural supercapacitors were tested on a two-cell configuration in a Swagelok cell, by punching out a 10 mm disc from the fabricated structural supercapacitors, with chronoamperometry, cyclic voltammetry, electrochemical impedance spectroscopy, and galvanostatic charge-discharge (GCD) tests on a Gamry Potentiostat (Reference 3000, Gamry Instruments). Chronoamperometry tests were conducted on structural supercapacitors by applying 100 mV step voltage for 5 min followed by recording the discharging current for 15 min. CV tests were performed on structural supercapacitors using a sweep rate of 100 mV/s in the voltage window of 0 to +1.0 V. EIS tests were also performed to further investigate the interfacial electrochemical properties of the fabricated structural supercapacitors. In the Nyquist plot, the first *x*-intercept of the high-frequency semicircle segment corresponds to the equivalent series resistance. EIS tests were performed in a frequency window of 10^6 to 1 Hz and 10 mV sinusoidal voltage amplitude were applied at room temperature. GCD tests were performed under different current densities (0.5-5 mA/g) at a maximum voltage of 2.7 V. In-plane shear and impact tests, of the fabricated structural supercapacitors were performed on a tensile testing machine (Tira-2800, Tira Instruments, Germany) and impact tester (GT-7-45-HMH, Gotech, Taiwan) respectively. In-plane shear tests were performed on a 20cm x 2.5cm rectangular test coupons, as per ASTM standard D3518, with a crosshead speed of 1.5 mm/min and a gauge length of 15 cm. Impact tests were

performed on a 13cm x 1.3cm rectangular test specimens, as per ASTM standard D6110, by using a pendulum energy of 7.5J.

The structure of $FeSe_2$ HPs and $FeSe_2$ HPs deposited carbon fabrics were demonstrated by SEM (obtained by FEI Nova 200 Nanolab Dualbeam/FIB System and FEI Helios 650 Nanolab Dualbeam/FIB System) and TEM (obtained by Thermo Fisher Scientific Talos F200X S/TEM and Spectra 300 Aberration-Corrected S/TEM), housed in the Michigan Center for Materials Characterization (MC)[2]. Tomography dataset was acquired by tilting the sample from -70° to 70° at an interval of 2°. Reconstruction was carried out with a Weighted Back-Projection algorithm in Tomviz software.

PTA was operated using Malvern NanoSight NS300 (Malvern Panalytical, UK) and a 405 nm violet laser module was selected. PTA tests were carried out in the Nanotechnicum at the UM Biointerface Institute. The protocol for preparing the dispersion for EIS and CV tests was adding 0.2 mg/mL $FeSe_2$ HPs and 0.1 M LiTf in DMF. The bulk electrolysis experiment requires a more concentrated dispersion so a suspension containing 0.3 mg/mL $FeSe_2$ HPs and 0.1 M LiTf in DMF was applied. Due to the limitation of the required instrument, 0.05 mg/mL $FeSe_2$ HPs were dispersed in ultra-purified water for PTA experiments.

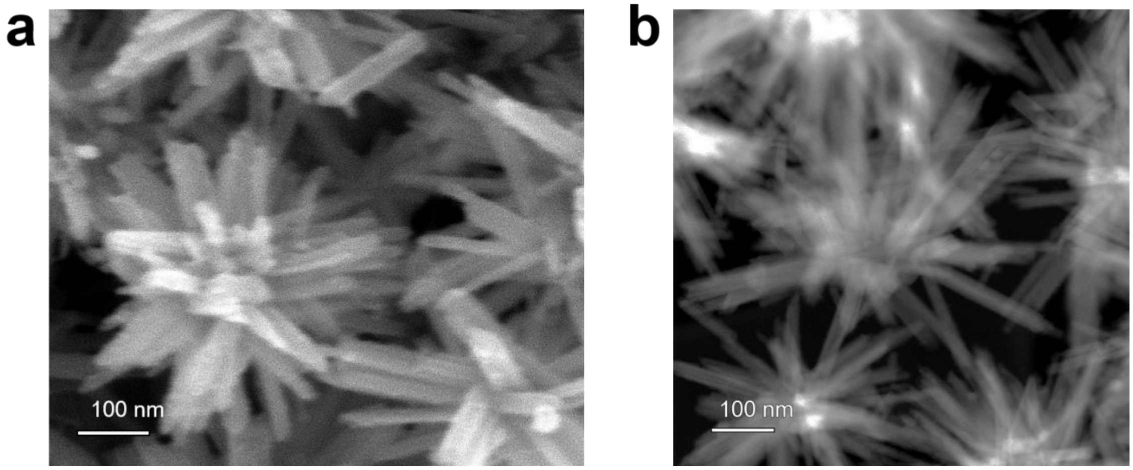

Figure S1. (a) SEM and (b) TEM image of $FeSe_2$ HPs.

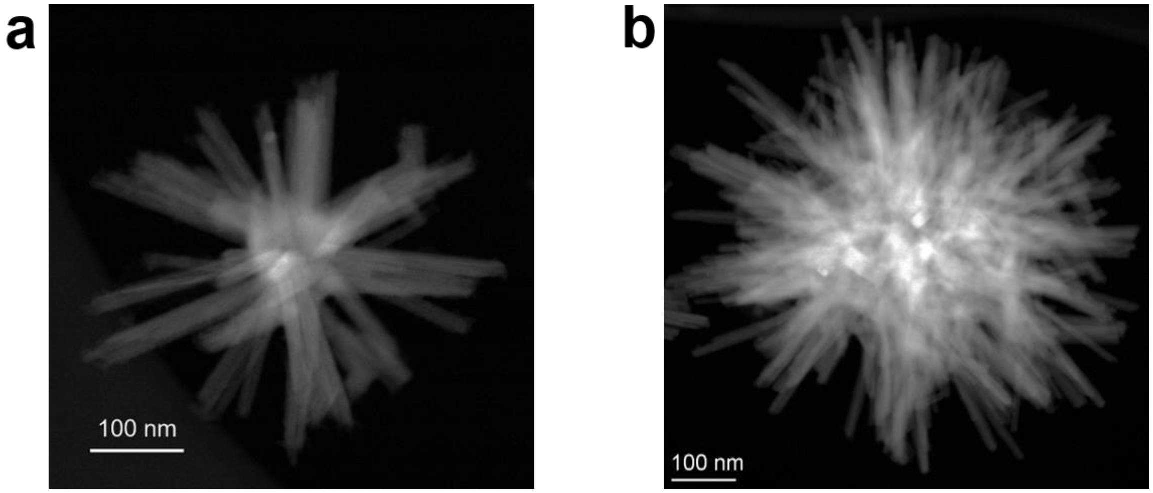

Figure S2. HAADF-STEM image of (a) coarse and (b) dense $FeSe_2$ HPs.

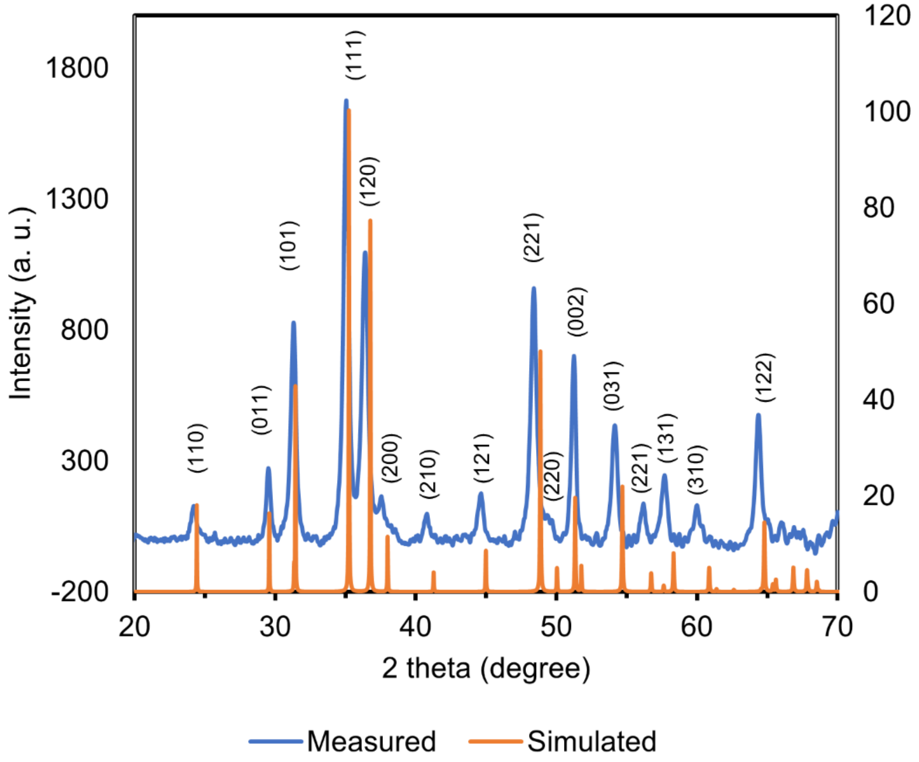

Figure S3. XRD patterns of synthesized $FeSe_2$ HPs and theoretically simulated spectra.

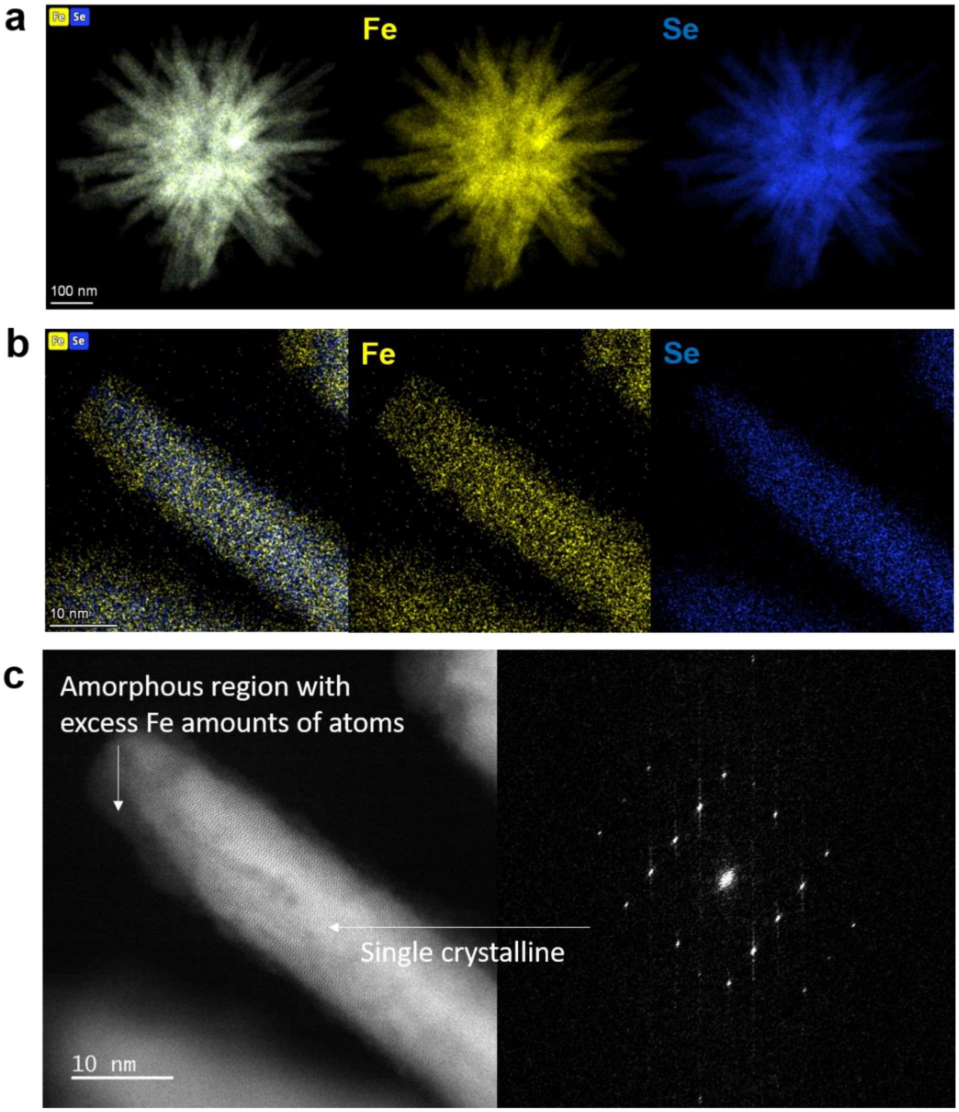

Figure S4. EDS mapping for the (a) $FeSe_2$ HP and (b) its single spike. (c) HAADF-STEM image showing an amorphous region at the outer edge of single crystalline $FeSe_2$. The FFT image was generated from the inner portion of the spike showing a single crystalline pattern.

1.7 First-principles calculations

The calculations were performed by using the density functional theory (DFT) framework in a Vienna ab initio simulation program (VASP)[3–5]. $FeSe_2$ had a Pnnm space group with the lattice parameter a = 3.58 Å, b = 4.79 Å and c = 5.74 Å for one formula unit per unit cell. $FeSe_2$ crystal structures with a 2×2×1 supercell was built to perform the optimization and properties calculations.

The conclusions were further validated by repeated calculations using a 2×1×1 supercell and 1×1×1 supercell. We employed the generalized gradient approximation (GGA) with the Perdew−Burke−Ernzerhof for solid (PBEsol). Full geometry optimization was performed until reaching the convergence criteria: the Hellmann−Feynman force component on each atom was less than 0.001 eV/Å and total energy was less than 1×10^{-8} eV. The effects of charging were simulated through the excess charge doping on $FeSe_2$ supercell. We employed a kinetic energy cutoff of 600 eV and an automatically generated 4 × 3 × 4 Monkhorst−Pack set of k points. The pseudopotentials were constructed by the electron configurations as Fe $3d^6$ $4s^2$ states and Se $4s^2$ $4p^4$ states. The phonon dispersion, IR, and other lattice vibration properties were carried out using DFPT methods through the phonopy[6] and phono3py[7] packages.

2. Determination of particle concentration of $FeSe_2$ Hedgehog Particles (HPs)

2.1 Particle tracking analysis (PTA)

PTA was applied to determine the particle concentration of $FeSe_2$ hedgehog particles (HPs) by counting particles.

For counting particles, generally there are two categories, namely ensemble methods and single particle counting.[8] Light absorption is the most universal ways but it requires an accurate determination of the molar extinction coefficient, which can be used even for nanoscale particles, for instance, gold[9,10], silver[11], and semiconductor NPs[12]. Dynamic light scattering (DLS) based on the Rayleigh scattering which is only practical for particles smaller compared to the wavelength of the incident light.

PTA is based on laser light scattering microscopy that can visualize particles during the Brownian motion. The movement of each particle is recorded by a camera that allows one to track and analyze each particle in real time. A laser beam illuminates a particle suspension at a low angle and the scattered light by particles in the liquid is collected by a conventional optical microscope.

By injecting HP dispersion slowly into the flow chamber, the PTA system can detect the light scattered from particles and interpret the concentration and size distribution after setting proper focus and threshold. Note that the gasket component and tubing of PTA flow cell allows only aqueous solutions to be injected into the PTA cells; non-aqueous solvents such as DMF are strictly prohibited.

2.2 Calculation of particle concentration and averaged molar mass of $FeSe_2$ HPs

Calculating particle concentration and averaged molar mass of $FeSe_2$ HPs can help us to determine the number of charges stored per $FeSe_2$ and per HP.

The concentration of HPs was measured by PTA because this method is very suitable for colloidal particles in size range from nanometers to microns. HPs are also convenient for this technique due to their high refractive index. Due to the high sensitivity of the technique and optical thresholds of the instrument, a highly diluted solutions are used. Thus, for PTA tests, we dispersed 0.75 mg $FeSe_2$ HPs in 15 mL ultra-purified water. Note that $FeSe_2$ HPs were in water instead of DMF due to the instrumental limitation, but it does not affect the result in determination of particle concentration. Here, c_{FeSe2} HPs = 0.75 mg/15 mL=0.05 mg/mL.

From PTA measurements, one can get the particle concentration is $(6.6 \pm 0.11) \times 10^8$ particle/mL, which translates into $\frac{((6.6 \pm 0.11) \times 10^8)\ \text{particle/mL}}{6.02 \times 10^{23}\text{particle/mol}}$ = $(1.1 \pm 0.018) \times 10^{-15}$ mol cm^{-3}

Therefore, the particle concentration of a 0.2 mg/mL $FeSe_2$ HPs dispersion is $4 \times \frac{((6.6 \pm 0.11) \times 10^8)\ \text{particle/mL}}{6.02 \times 10^{23}\text{particle/mol}}$ = $(4.4 \pm 0.076) \times 10^{-15}$ mol cm^{-3}

So, the averaged molar mass of HP ($M_{FeSe2\ HP}$) is

$$M_{FeSe2\ HP} = \frac{0.2\ \text{mg cm}^{-3}}{4.4 \times 10^{-15}\text{mol cm}^{-3}} = 4.6 \times 10^{10}\text{g mol}^{-1}$$

3. Electrochemical properties of $FeSe_2$ HPs in dispersion

3.1 Electrochemical Impedance Spectroscopy (EIS) and Nyquist plot of $FeSe_2$ HPs in DMF

The fundamental approach of all impedance methods is to apply a small amplitude sinusoidal excitation signal to the system under investigation and measure the response (current, voltage, *etc.*). A low amplitude sine wave $\Delta E\, sin(\omega t)$, of a particular frequency ω, is superimposed on the DC polarization voltage E_0. This results in a current response of a sine wave superimposed on the DC current $\Delta i\, sin(\omega t + \varphi)$. The current response is shifted with respect to the applied potential. The Taylor series expansion for the current is given by:

$$\Delta i = \left(\frac{di}{dE}\right)_{E_0,i_0} \Delta E + \frac{1}{2}\left(\frac{d^2 i}{dE^2}\right)_{E_0,i_0} \Delta E^2 + \cdots$$

If the magnitude of the perturbing signal ΔE is small, then the response can be considered linear in first approximation. The higher order terms in the Taylor series can be assumed to be negligible. The impedance of the system can then be calculated using Ohm's law as: $Z(\omega) = E(\omega)/i(\omega)$. Characteristic $Z(\omega)$ is referred to as impedance; it is a complex quantity with a magnitude and a phase shift which depends on the frequency of the applied potential ω. In Cartesian coordinates, the impedance is given by: $Z(\omega) = Z'(\omega) - jZ''(\omega)$ where $Z'(\omega)$ is the real part of the impedance and $Z''(\omega)$ is the imaginary part and $j = \sqrt{-1}$. The plot of the real part of impedance against the imaginary part is typically referred to as Nyquist plot. While plotting data in the Nyquist format the real axis must be equal to the imaginary axis so as not to distort the shape of the curve. The shape of the curve is important in making qualitative interpretation of the data.[13] Here, a clear semicircle was observed in the Nyquist plot (**Figure S5**).

A typical electrochemical impedance experimental setup consists of an electrochemical cell, a potentiostat, and a frequency response analyzer that applies the sine wave and analyze the response of the system to determine the impedance of the system. We chose the most common – three-electrode system for EIS test. The impedance is measured between the reference electrode and the working electrode (S).[14] In a typical CV experiment using a 3-mm disk electrode, the current response is usually in the magnitude of 10^{-6} A.

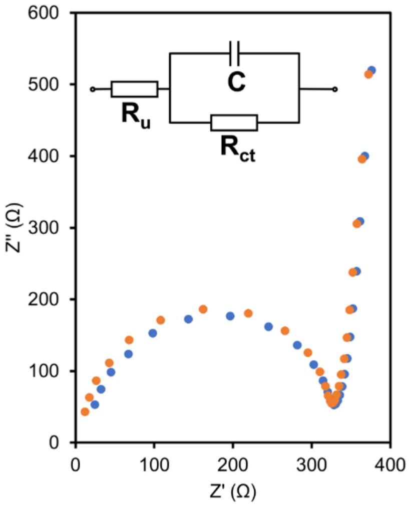

Figure S5. Nyquist plot of 0.2 mg/mL $FeSe_2$ HPs in DMF with 0.1 M LiTf. Frequency sweep from 700 kHz to 1 Hz. Data from 700 kHz to 554 Hz are shown in the plot. The Randles circuit is chosen as the equivalent circuit.

As for the data analysis, we focus on resistance mostly. The potential drop between the reference electrode and the working electrode, is the ohmic resistance, or the uncompensated resistance. The uncompensated resistance (R_u) depends on the conductivity of the electrolyte and the geometry of the electrode. In a Nyquist plot, the intersection of the impedance data with the real part of the axis at the high frequency end gives the uncompensated resistance.[15] From **Figure S5**, an uncompensated resistance (R_u) was 18 ± 6 Ω. Therefore, the *iR* compensation of 0.02 mV can be safely neglected. Moreover, Z' (R_{ct}) = 308 ± 5 Ω, Z'' (C) = 182 ± 4 Ω.

3.2 Illustration of reaction of $FeSe_2$ HPs in dispersion

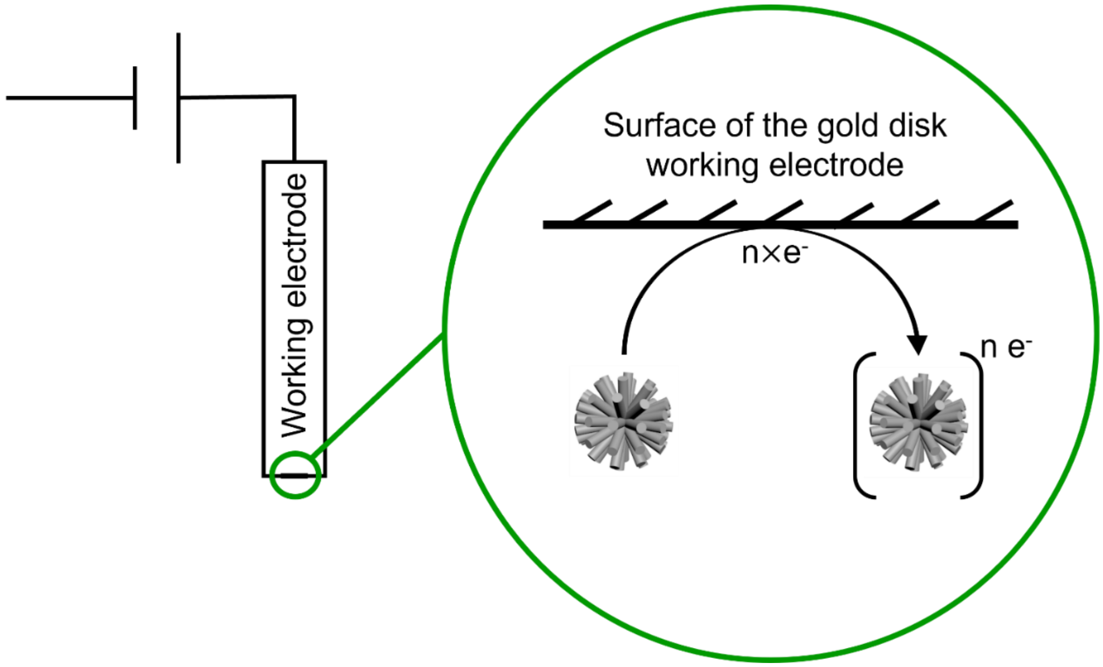

Figure S6. Illustration of a $FeSe_2$ HP been charged on the surface of the working electrode.

3.3 Cyclic Voltammograms (CV) of $FeSe_2$ HPs in DMF

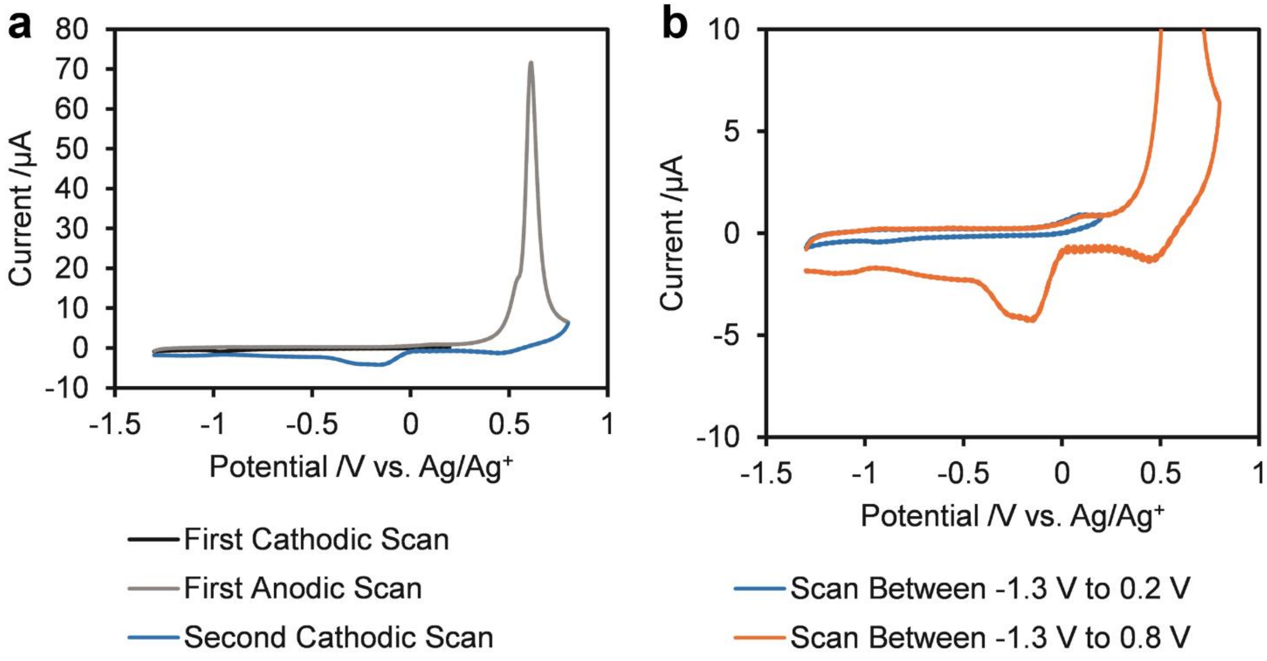

Figure S7. CVs of 0.2 mg/mL $FeSe_2$ HPs in DMF with 0.1 M LiTf. (a) Initially scanned in the cathodic direction from 0.2 V to -1.3 V (black), along with two half cycles from -1.3 V to 0.8 V (gray) and back to -1.3 V (blue). Scan rate of 100 mV/s. (b) Blue: -1.3 V to 0.2 V, the fifth cycle; Orange: the first anodic scan and the second cathodic scan in (a).

4. Calculation of charging capacity

4.1 Charging capacity of a single $FeSe_2$ HP

From PTA experiments, $M_{FeSe2\,HP} = 4.6 \times 10^{10}\ g/mol$. Because $M_{FeSe2} = 213.77\ g/mol$

$$N_{FeSe2} = 4.6 \times 10^{10}/213.77 = 2.2 \times 10^{8}$$

Therefore, an average of 2.2×10^8 $FeSe_2$ crystalline unit cells are in a single HP. From bulk electrolysis experiment results (**Figure 3A**),

$$n = \frac{q/F}{c_{FeSe2} \times V/M_{FeSe2}} = 0.7$$

an average of 0.7 charges could be stored per $FeSe_2$ crystalline unit cell.

Thus, the number of charges stored per $FeSe_2$ HPs will be

$$N_{HP} = 0.7 \times 2.2 \times 10^{8} = 1.5 \times 10^{8}$$

4.2 Gravimetric estimation of $FeSe_2$ HPs

Gravimetric measurements can be used to calculate the particle concentration and the averaged molar mass of $FeSe_2$ HPs, proving the accuracy of results from PTA experiments. Moreover, the volume of a single HP can be applied in the calculation comparing the charging capacity of $FeSe_2$ HPs with other previously studied semiconductor nanoparticles.

Assume that the density of $FeSe_2$ nanospikes and $FeSe_2$ spherical core are the same as for bulk $FeSe_2$[16].

$$\rho_{FeSe2\,HPs} = \rho_{FeSe2} = 7.14\ Mg\ m^{-3} = 7.14\ mg\ mm^{-3}$$

From SEM images, the mean diameter of HP D_{HP} = 556 nm, the mean length of nanospikes is l_{spike} = 276 nm. We decided to use TEM images (**Figure S8a**) to calculate the diameter of nanospikes due to their smaller dimension. From the statistical analysis (**Figure S8b**) of TEM images, the diameter of nanospikes D_{spike} = 21 ± 5 nm.

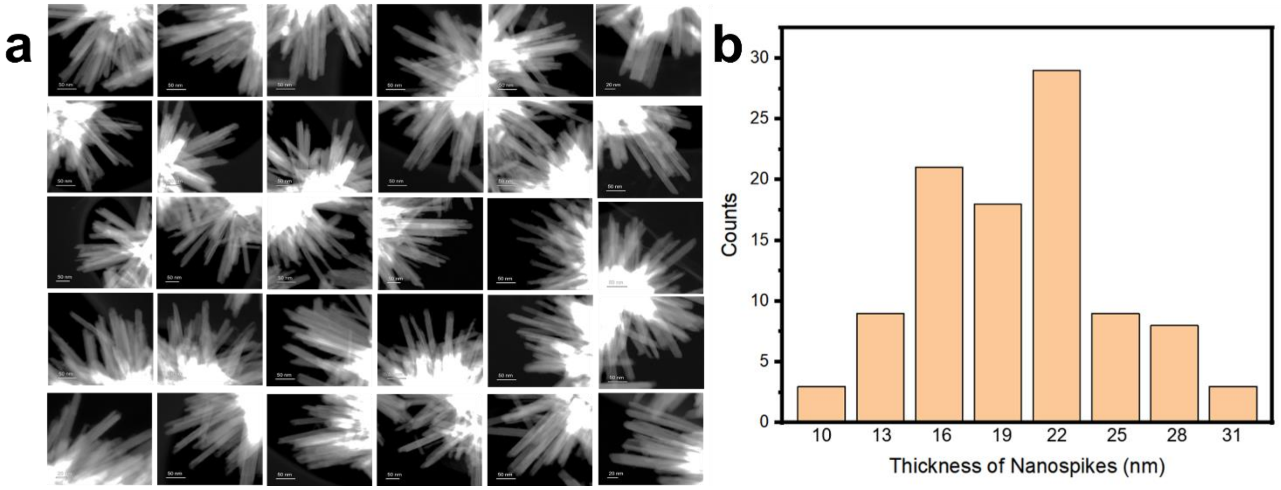

Figure S8. (a) TEM images used to estimate the diameter of nanospikes. (b) Histogram of the thickness of nanospikes (nm).

The diameter of the $FeSe_2$ spherical core D_{core} is

$$D_{core} = D_{HP} - 2l_{spike} = 556 - 276 \times 2 = 4 \text{ nm}$$

The volume of the $FeSe_2$ spherical core V_{core} is

$$V_{core} = \frac{4}{3}\pi\left(\frac{D_{core}}{2}\right)^3 = \frac{4}{3}\pi\left(\frac{4}{2}\right)^3 = 34 \text{ nm}^3$$

From SEM images, about 100 spikes are visible. Assuming that there are 100 spikes on the other side, which is invisible from the image, so there is a total of 200 nanospikes on a single HP.

We think that the cross-sectional of nanospike is rhombus shaped from the TEM images. The nanospikes are thicker in the middle, and thinner in the edges. The structure is not hollow by looking at

the STEM images. Therefore, we believe that the nanospikes are like blade. The average volume of one spike is

$$V_{spike} = l_{spike} \times \frac{1}{2} D_{spike}^2 = 276 \times \frac{1}{2} \times 21^2 = 6.1 \times 10^4 nm^3$$

The volume $FeSe_2$ phase in a single HP is

$$V_{HP} = V_{core} + 200 V_{spike} = 34 + 200 \times 6.1 \times 10^4 = 1.2 \times 10^7 nm^3 = 1.2 \times 10^{-11}\ \mathrm{mm}^3$$

The mass of a single HP (m_{HP}) is

$$m_{HP} = V_{HP} \times \rho_{FeSe2\ HP} = 1.2 \times 10^{-11} \times 7.14 = 8.7 \times 10^{-11}\ \mathrm{mg}$$

Therefore, the number of particles in 0.05 mg $FeSe_2$ is

$$N_{HP} = 0.05/8.7 \times 10^{-11} = 5.8 \times 10^8$$

The particle concentration in the PTA experiment is going to be

Particle concentration = 5.8×10^8 particle/mL = $\frac{5.8\times10^{8}\mathrm{particle/mL}}{6.02\times10^{23}\mathrm{particle/mol}}$ = 9.6×10^{-16} mol cm^{-3}

Their averaged molar mass is

$$M_{FeSe2\ HP} = \frac{0.05\ \mathrm{mg\ cm^{-3}}}{9.6 \times 10^{-16} \mathrm{mol\ cm^{-3}}} = 5.2 \times 10^{10} \mathrm{g\ mol^{-1}}$$

The averaged molar mass of $FeSe_2$ HP calculated by the gravimetric method is close to the value measured by PTA experiments, demonstrating the accuracy of results from PTA experiments.

4.3 Comparison of charges per volume of a single particle

For a $FeSe_2$ HP, the number of charges is 1.5×10^8.

$$volume = V_{HP} = 1.2 \times 10^7 nm^3$$

$$\frac{charge}{volume} = \frac{1.5 \times 10^8}{1.2 \times 10^7} = 13\ e^-/nm^3$$

For a CdS nanoparticle[17], the number of charges is 50.

$$volume = \frac{4}{3}\pi\left(\frac{D}{2}\right)^3 = \frac{4}{3}\pi \times \left(\frac{4}{2}\right)^3 = 34\ nm^3$$

$$\frac{charge}{volume} = \frac{50}{34} = 1.5\ e^-/nm^3$$

For a CdS colloidal semiconductor particle[18], the number of charges is 270.

$$volume = \frac{4}{3}\pi\left(\frac{D}{2}\right)^3 = \frac{4}{3}\pi \times \left(\frac{8}{2}\right)^3 = 268\ nm^3$$

$$\frac{charge}{volume} = \frac{270}{268} = 1\ e^-/nm^3$$

Table S1. Comparison of charges per volume of a single particle.

Particles	$FeSe_2$ HP	CdS Nanoparticle	CdS Colloidal Semiconductor Particle
Charges per volume (e^-/nm^3)	13	1.5	1

4.4 Comparison of charges per surface area of a single particle

For a $FeSe_2$ HP, the number of charges is 1.5×10^8.

$$Surface\ area = core + spikes = \pi D_{core}^2 + 200 \times 2\sqrt{2} D_{spike} \times L_{spike}$$

$$= \pi \times 4^2 + 200 \times 2\sqrt{2} \times 21 \times 276 = 3.3 \times 10^6 nm^2$$

$$\frac{charge}{area} = \frac{1.5 \times 10^8}{3.3 \times 10^6} = 46\ e^-/nm^2$$

For a CdS nanoparticle[17], the number of charge is 50.

$$surface\ area = \pi D^2 = \pi \times 4^2 = 50\ nm^2$$

$$\frac{charge}{area} = \frac{50}{50} = 1\ e^-/nm^2$$

For a CdS colloidal semiconductor particle[18], the number of charges is 270.

$$surface\ area = \pi D^2 = \pi \times 8^2 = 201\ nm^2$$

$$\frac{charge}{area} = \frac{270}{201} = 1.3\ e^-/nm^2$$

Table S2. Comparison of charges per surface area of a single particle.

Particles	$FeSe_2$ HP	CdS Nanoparticle	CdS Colloidal Semiconductor Particle
Charges per surface area (e^-/nm^2)	46	1	1.3

4.5 Comparison of charges per crystal unit cell for $FeSe_2$ HP and quantum dots

According to *Electrochemical Methods Fundamentals and Applications Second Edition* page 231[19], the peak current in CV curves, for reversible reactions can be represented as

$$i_p = (2.69 \times 10^5) n^{3/2} A D_O^{1/2} C_O^* v^{1/2};$$

while the peak current for irreversible reactions (page 236)

$$i_p = (2.99 \times 10^5) \alpha^{1/2} A D_O^{1/2} C_O^* v^{1/2}.$$

In the CV system of $FeSe_2$ HPs, the number of electron transfer $N_{HP} = 1.5 \times 10^{8}$, the surface area of the working electrode $A = 0.071 cm^{2}$, the diffusion coefficient (obtained by Dynamic Light Scattering experiments) $D = 7.93 \times 10^{-9}\ cm^{2} s^{-1}$, the bulk concentration of the active species $C^{*} = 4.4 \times 10^{-15} mol\ cm^{-3}$, scan rate $v = 0.1\ V/s$. For most irreversible reactions, α is between 0.3 to 0.7, and thus we can assume that $\alpha \sim 0.5$.

Comparing the theoretical peak currents to experimental ones, $i_{p,reversible} \sim 4.3 \times 10^{-3} A$ and $i_{p,irreversible} \sim 1.9 \times 10^{-15} A$ assuming that HP undergoes an oxidation reaction (O1) reversibly or irreversibly, respectively. In the actual CV experiments, $i_p \sim 3.5 \times 10^{-5} A$ (**Figure 2A**). Therefore, the reversible equation ($i_p = (2.69 \times 10^{5}) n^{3/2} A D_O^{1/2} C_O^{*} v^{1/2}$) is the better equation to estimate the peak current for CV system of $FeSe_2$ HPs.

In the CV system of CdS nanoparticles[17], $i_p = 2 \times 10^{-6} A$, $MW_{CdS} = 144.46\ g/mol$, $A = 0.062 cm^{2}$, $n = 50$, $D = \frac{kT}{3\pi\eta d(H)} = 1.2 \times 10^{-6} cm^{2} s^{-1}$, $v = 0.05\ V/s$. By using the same equation as used in the CV system of $FeSe_2$ HPs,

$$C^{*} = \frac{i_p}{(2.69 \times 10^{5}) n^{3/2} A D^{1/2} v^{1/2}} = \frac{2 \times 10^{-6}}{2.69 \times 10^{5} \times 50^{3/2} \times 0.062 \times \sqrt{1.2 \times 10^{-6}} \times \sqrt{0.05}}$$

$$= 1.4 \times 10^{-9} mol/cm^{3}$$

So, the CdS nanoparticle concentration is $1.4 \times 10^{-9} mol/cm^{3}$.

$$c = \frac{1 \times 10^{-3} g/cm^{3}}{144.46\ g/mol} = 6.92 \times 10^{-6}\ mol/cm^{3}$$

Thus, the CdS crystalline unit cell concentration is $6.92 \times 10^{-6} mol/cm^{3}$

Dividing c by C^{*}, we can get that there is an average of 5016 CdS units per CdS nanoparticle.

The charge per unit cell for CdS nanoparticle is 50/5016 = 0.01, which is less than the charging capacity of 0.7 per $FeSe_2$.

4.6 Calculation of charge stored for a metal particle

Assumptions: diameter $D = 1\ \mu m$, potential difference $E = 0.8\ V$ (from OCP of -0.1 V to 0.7 V vs. Ag/Ag^+). The double layer capacitance (C_d) of electrode-solution interface is typically in the range of 10 to 40 $\mu F/cm^2$ (assume 10 $\mu F/cm^2$ in calculation).[19]

The charge stored on the metal particle per surface area $q = C_d E = 8\ \mu C/cm^2$

The surface area of a 1 μm metal particle $A = \pi D^2 = \pi(10^{-6})^2 = 10^{-12}\pi\ m^2 = 10^{-8}\pi\ cm^2$

The charge stored on the metal particle $Q = qA = 8\pi \times 10^{-14} C$

Because one charge has $1.6 \times 10^{-19} C$,

Therefore, the number of charges $N = \frac{8\pi\times10^{-14}}{1.6\times10^{-19}} = 1.6 \times 10^6$

5. Structural characterization of the highly charged states on $FeSe_2$ HPs

5.1 X-ray Photoelectron Spectroscopy of Fe and Se on $FeSe_2$ HPs upon charging

XPS of various $FeSe_2$ HPs helps us understand the valence changes during electrochemical measurements. Se 3d XPS spectra in **Figure S9** displayed a peak at 57.6 eV that was attributed to Se atoms bonded with oxygen (Se-O bond); it corresponds to the surface oxidation state of selenium species ascribed to the high surface activities as building blocks commonly seen in many 3D $FeSe_2$ nanostructure.[20] The other two peaks in XPS spectra at 54.0 eV ($3d_{3/2}$) and 53.1 eV ($3d_{5/2}$) originated from spin orbit splitting Se 3d states with peak separation of less than 1 eV.

When the $FeSe_2$ HPs were charged to 0.7 V vs. Ag/Ag^+ in the bulk electrolysis experiment, both Se $3d_{3/2}$ and Se $3d_{5/2}$ peaks shifted about 2.5 eV higher in binding energy, meaning that selenium atoms will be oxidized during the bulk oxidation. Because these peaks are similar in binding energy to the Se-O peak, it was not clear if Se-O still existed. It is likely that selenium atoms are oxidized to a similar oxidation state as selenium species in the Se-O peak.

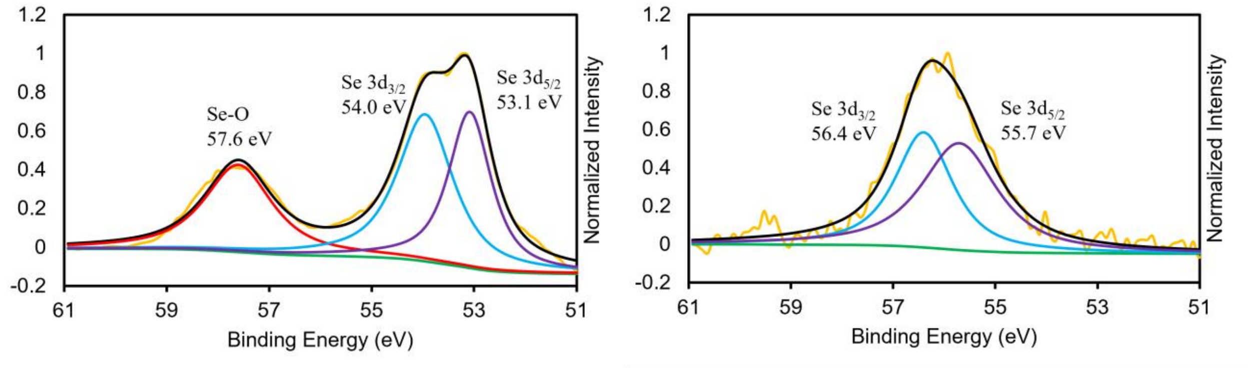

Figure S9. Se 3d XPS spectra of $FeSe_2$ HPs (left) and $FeSe_2$ HPs charged to OCP of 0.7 V vs. Ag/Ag^+ by bulk electrolysis experiments (right). Legends: Black - peak sum; Green - background; Red - peak 1 (Se-O); Blue - peak 2 (Se $3d_{3/2}$); Purple - peak 3 (Se $3d_{5/2}$); Yellow - raw intensity.

Original Fe 2p XPS spectrum (**Figure S10**) contained four peaks, including signature $2p_{1/2}$ (718.4 eV) and its satellite peak (723.9 eV) and $2p_{3/2}$ (705.5 eV) peak and its satellite peak (710.0 eV). The spin separation of Fe 2p was 12.9 eV, exhibiting characteristics of divalent iron.[21] The satellite peaks may indicate the existence of a higher oxidation state of iron present in the $FeSe_2$ HPs.[22] For Fe 2p spectra obtained upon charging $FeSe_2$ HPs to 0.7 V vs. Ag/Ag^+ during bulk electrolysis experiments compared to the original spectrum, the Fe $2p_{3/2}$ peak and its satellite peak shifted to the higher binding energy direction for 2 eV but the Fe $2p_{1/2}$ peak did not shift. Therefore, the peak separation reduced to

10.7 eV. This may indicate the oxidation of iron atoms due to the presence of divalent iron in $FeSe_2$ HPs.

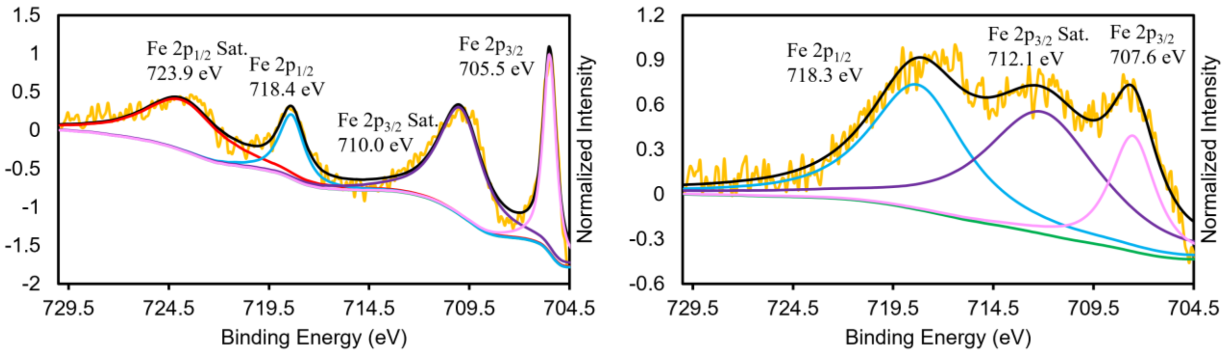

Figure S10. Fe 2p XPS spectra of $FeSe_2$ HPs (left) and $FeSe_2$ HPs charged to OCP of 0.7 V vs. Ag/Ag^+ by bulk electrolysis experiments (right). Legends: Black - peak sum; Green - background; Red - peak 1 (Fe $2p_{1/2}$ sat.); Blue - peak 2 (Fe $2p_{1/2}$); Purple - peak 3 (Fe $2p_{3/2}$ sat.); Pink – peak 4 (Fe $2p_{3/2}$); Yellow - raw intensity.

5.2 X-ray diffraction (XRD) of $FeSe_2$ with different amounts of excess charges

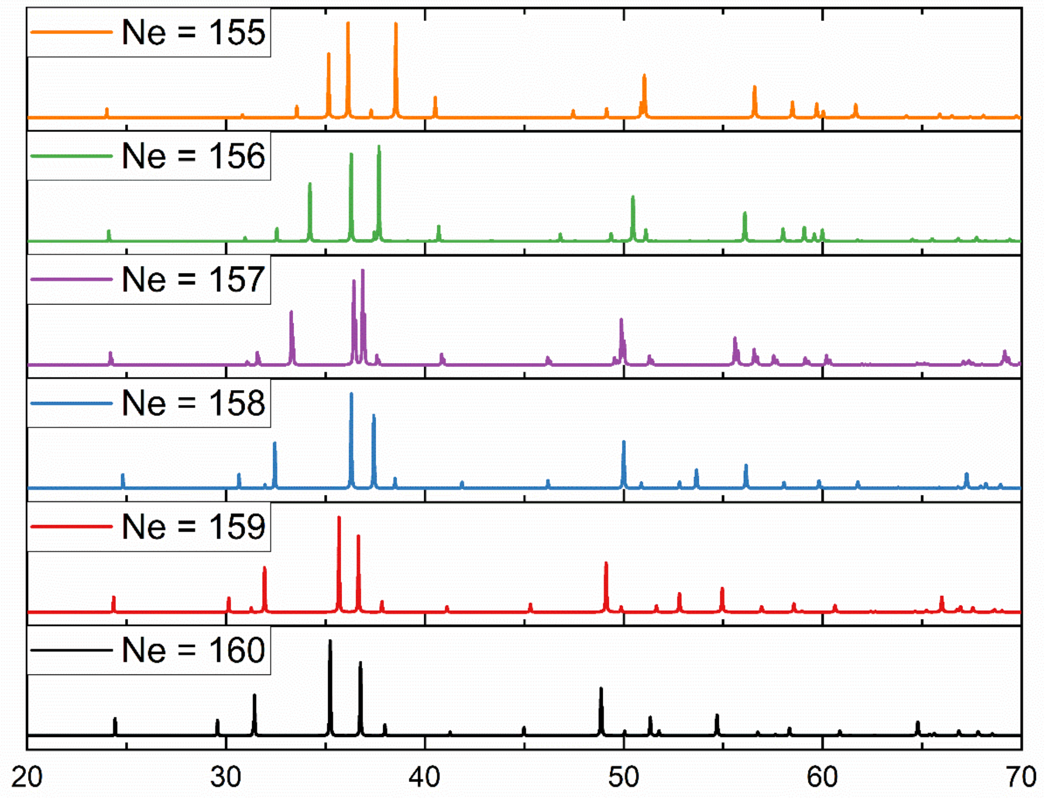

Figure S11. XRD plots comparison between $FeSe_2$ supercell adding 0 (N_e = 160, uncharged state) to 5 charges (N_e = 155).

Upon charging process, peaks including (011), (101), (120), (121), (211), and (031) shifted gradually to larger degrees, and peaks including (111), (131) and (310) shifted slightly to the larger degrees, while the (200) peak remained in the same position. In the case of $FeSe_2$ supercell with five positive charges introduced, the (200) peak emerged between the (111) and (120) peak, while the (122) peak disappeared.

5.3 Selenium atoms displacement with different amounts of excess charges

Table S3. Selenium atoms displacement upon charging.

Valence Electrons (a.u.)	Se1			Se3			Se4			Se5		
	x_{Se1}	y_{Se1}	z_{Se1}	x_{Se3}	y_{Se3}	z_{Se3}	x_{Se4}	y_{Se4}	z_{Se4}	x_{Se5}	y_{Se5}	z_{Se5}
155	1	0.40429	0.64051	1	0.09571	0.35949	0.75	0.15429	0.85949	0.75	0.34571	0.14051
156	1	0.3969	0.63446	1	0.1031	0.36554	0.75	0.15477	0.85836	0.75	0.34524	0.14164
157	1	0.39797	0.63787	1	0.10203	0.36213	0.75	0.14794	0.86216	0.75	0.35207	0.13784
158	1	0.39383	0.63541	1	0.10617	0.36459	0.75	0.14383	0.86459	0.75	0.35617	0.13541
159	1	0.39271	0.63452	1	0.10729	0.36547	0.75	0.14271	0.86548	0.75	0.35729	0.13452
160	1	0.39038	0.63359	1	0.10963	0.36641	0.75	0.14037	0.8664	0.75	0.35963	0.13359

The relaxation ratio mentioned in **Figure 4E** defines the atomic displacement.

$$\Delta y_i = \frac{y_i - y_0}{y_0} \times 100\%$$

$$\Delta z_i = \frac{z_i - z_0}{z_0} \times 100\%$$

Where y_i is the fractional coordinate of atom along [010] direction, z_i is the fractional coordinate of atom along [001] direction.

5.4 Lattice thermal conductivity of $FeSe_2$ upon charging

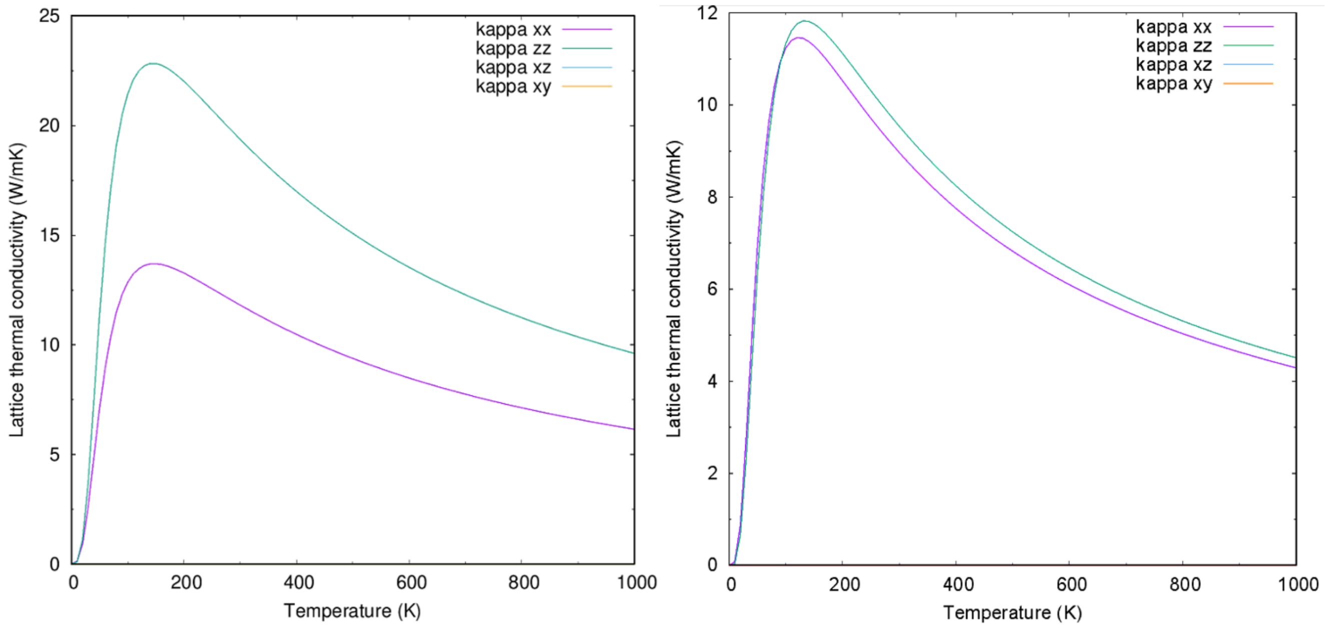

Figure S12. Lattice thermal conductivity of uncharged (left) and charged (right) $FeSe_2$, calculated from relaxation time approximation (RTA) and linearized phonon Boltzmann transport equation.

5.5 Density of States (DOS) of Fe 3d orbital & Se 3p orbital with different amounts of excess charges

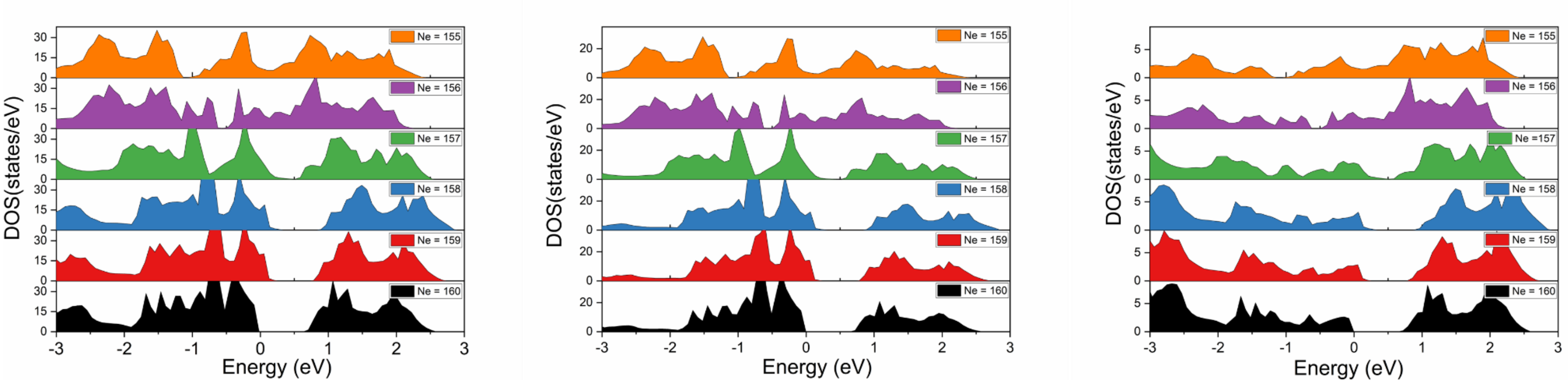

Figure S13. Total and partial DOS of $FeSe_2$ supercell (left panel), Fe 3d orbital (middle panel) and Se 3p orbital (right panel) with different amounts of excess charges (From N_e =160 [uncharged] to N_e = 155).

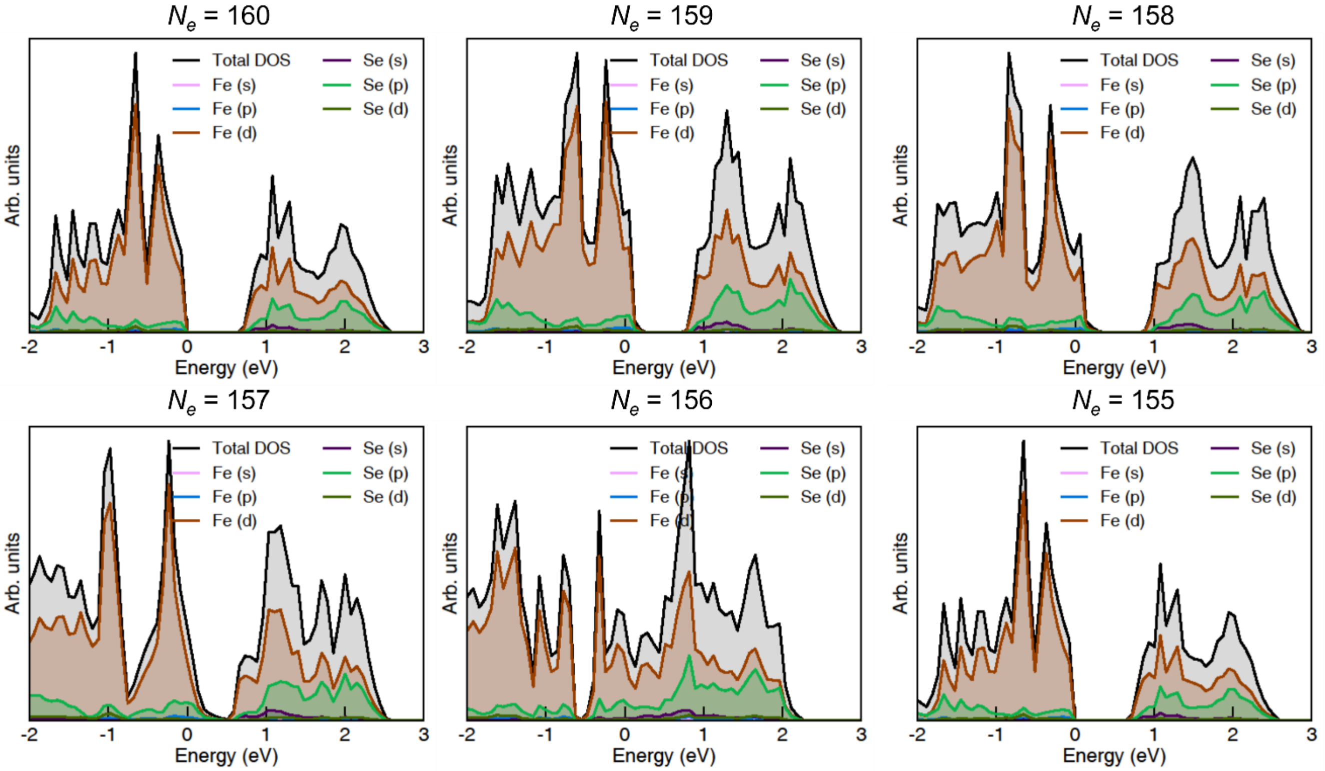

Figure S14. Partial DOS of Fe and Se atoms. The conduction band minimum (CBM) shifted to lower energy from N_e=160 to N_e=156.

6. Application of $FeSe_2$ HPs in structural supercapacitor

6.1 Comparison of structural supercapacitors with the literature

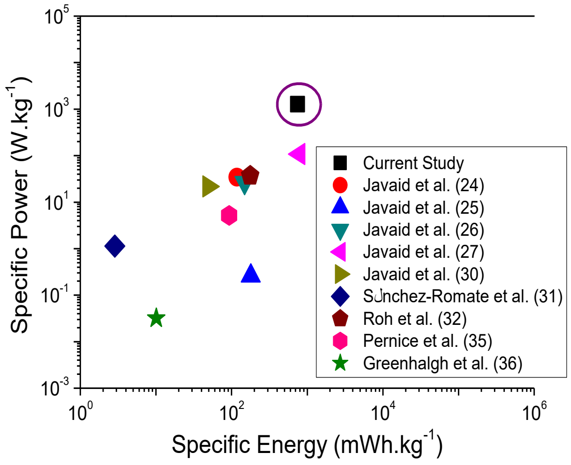

Figure S15. Ragone plot comparing the specific energy and power of the structural supercapacitor devices, reported in current study, with the devices reported in literature.

Table S4. Comparison of the electrochemical and mechanical properties of the current study with other **structural supercapacitors reported** in the literature available at the time of the final corrections.

Sr.	Paper	Electrode	Electrolyte	Electrochemical*				Mechanical	
				C_g	C_v	E	P	G_{12}	σ_{12}
				mF g^{-1}	mF cm^{-3}	mWh kg^{-1}	W kg^{-1}	GPa	MPa
**1**	**Current Study**	**CF/ $FeSe_2$ HPs**	**PEGDGE/1%Li/1%IL**	**77.6**	**107.1**	**744.7**	**1269**	**1.84**	**11.3**
2	Hudak et al. [23]	CF/MWCNTs	PEG-LiTf	125	--	17.4	--	2.90 (Flex.)	21 (Flex.)
3	Javaid et al. [24]	ACF	PEGDGE/10%IL/ MSP	--	120.4	117.7	34.4	1.76	38.2
4	Javaid et al. [25]	CF/Al-LiCoO2	DGEBA/20%Li	--	--	180	0.26	0.75	25.7
5	Javaid et al. [26]	CF/10GNP	DGEBA/40%Li-PC	--	118.7	148.2	26.4	3.07	21.1
6	Javaid et al. [27]	CF/CA.GNP5	DGEBA/50%Li-PC	353.7	79.8	786.05	107.8	2.64	14.7
7	Reece et al. [28]	ACF	Epoxy/TEABF	101.6	--	--	--	0.30 (Flex.)	29.1 (Flex.)
8	Ganguly et al.[29]	CF/ Graphene nanoflakes/Urea activation	PEGDGE/10%IL	47.98	56.14	0.066	0.788	23.3 (Tens.)	95 (Tens.)
9	Javaid et al. [30]	ACF/PAni-0.05	DGEBA/50%Li-PC	22.24	5.97	49.4	21.6	1.32	2.33

10	Sánchez-Romate et al. [31]	CF/GNPs	LY-PEGDGE-IL	9.6	--	2.86	1.139	--	--
11	Roh et al. [32]	CF/ nickel cobaltite nanowire	Polyester resin/ Li/IL	37.43	--	176.4	36.96	--	14.1
12	Subhani et al.[33]	CF/graphene aerogel (28%)	Epoxy/IL	56	--	--	0.023	--	--
13	Fang et al. [34]	CF/ rGO/CuO	Polymer cement electrolyte		221.5	0.6mWh/ cm^{-2})	0.6mW/ cm^{2}	--	--
14	Pernice et al.[35]	CF/CAG	Epoxy/IL	212	--	93	5.2	1.7	13.7
15	Greenhalgh et al.[36]	CF/CNT	Epoxy/IL/Li (4.6 mol L^{-1})	10.0	--	10.1	0.032	0.45	14.1
16	Javaid et al.[37]	ACF	PEGDGE/0.8%Li	--	0.41	0.51	--	0.39	7.34
17	Javaid et al. [38]	CF	PEGDGE/10%IL	--	7.06	8.82	--	0.51	9.77

* Electrochemical properties are based on the overall device weight and volume.

6.2 Electrochemical properties of macroscale tested structural supercapacitors

The electrochemical properties of macroscale structural supercapacitors were also tested after fabrication and the results are plotted in **Figure S16A-C**. The comparison of small scale (10 mm Swagelok cell) and macroscale (20cm x 20cm) structural supercapacitors is displayed in **Figure S16D-F**. Greenhalgh *et al.*[39] has previously reported a similar comparison and has attributed the decrease in electrochemical properties to the resistive losses, notably increased resistances of the current collection.

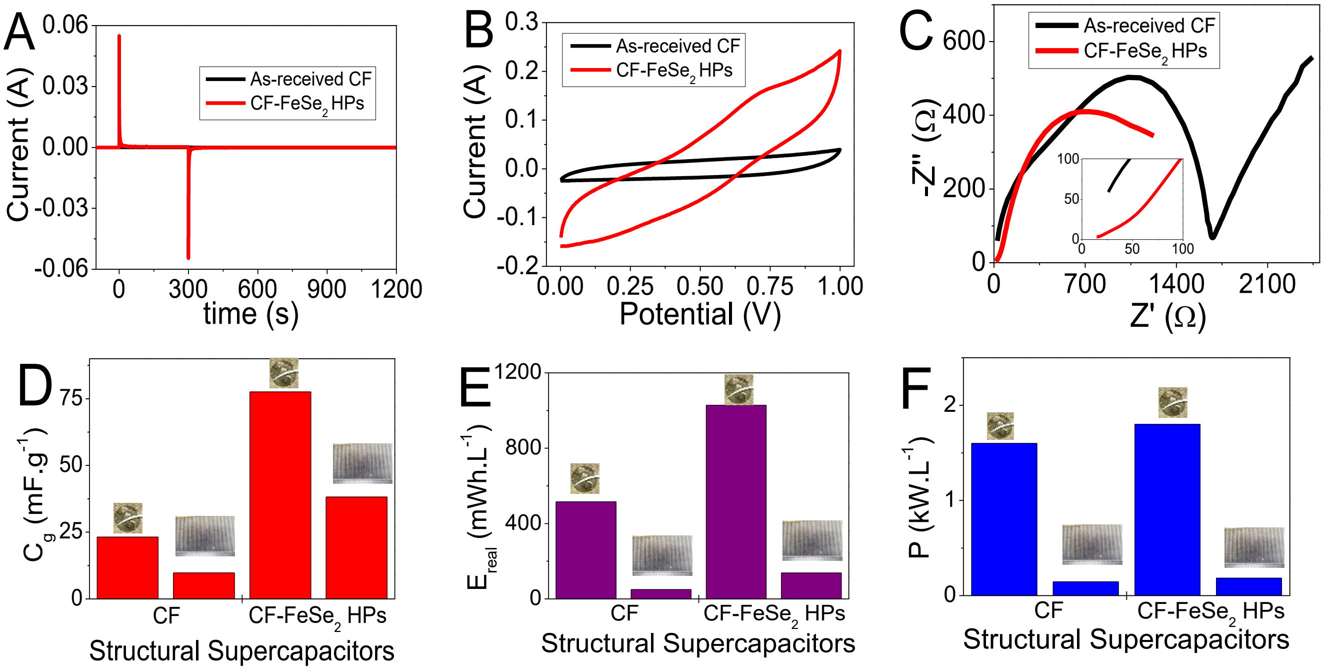

Figure S16. (A) Chronoamperometric charge-discharge test, (B) CV test at a scan rate of 100 mV s^{-1}, and (C) EIS test for macroscale A4 sized structural supercapacitors with as-received CF electrodes and CF-$FeSe_2$ HPs electrodes. Comparison of (D) gravimetric capacitance, (E) specific energy and (F) specific power between small scale (10 mm in Swagelok cell) and macroscale A4 sized structural supercapacitors.

6.3 Cyclic stability experiment of CF- $FeSe_2$ HP structural supercapacitor

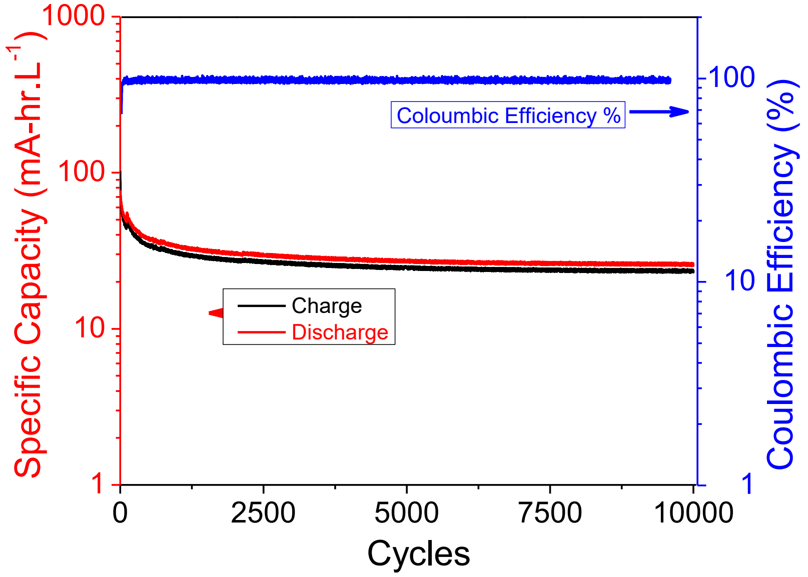

Figure S17. Charge/discharge capacities and Coulombic efficiencies versus the number of cycles for the structural supercapacitor with $FeSe_2$ HPs deposited carbon fabric electrodes. The tests are performed at a current density of 1 mA/g.

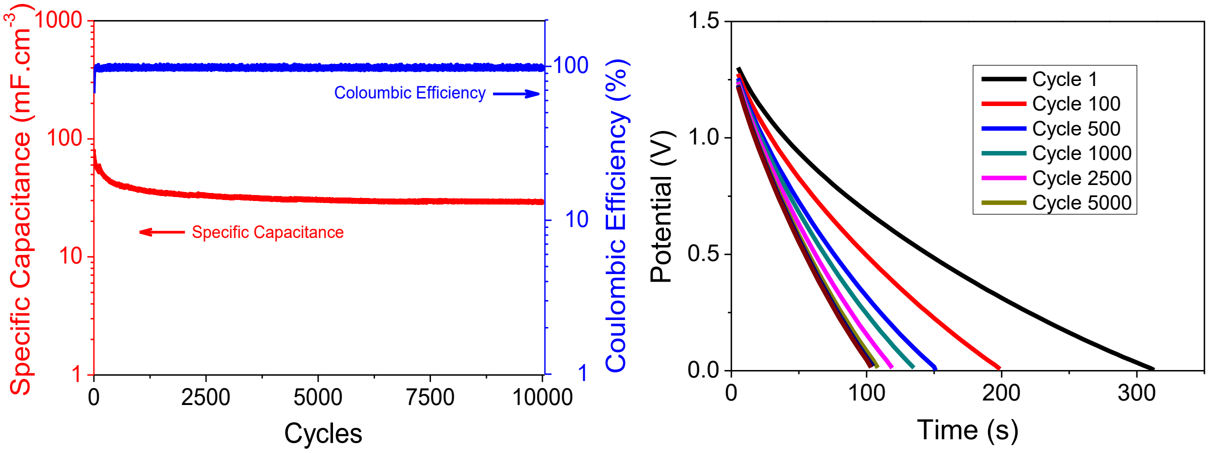

Figure S18. Left: specific capacitance and coulombic efficiency versus the number of cycles. Right: discharge plots at cycle 1, 100, 500, 1000, 2500, 5000 for the structural supercapacitor with $FeSe_2$ HPs deposited carbon fabric electrodes. The tests are performed at a current density of 1 mA/g.

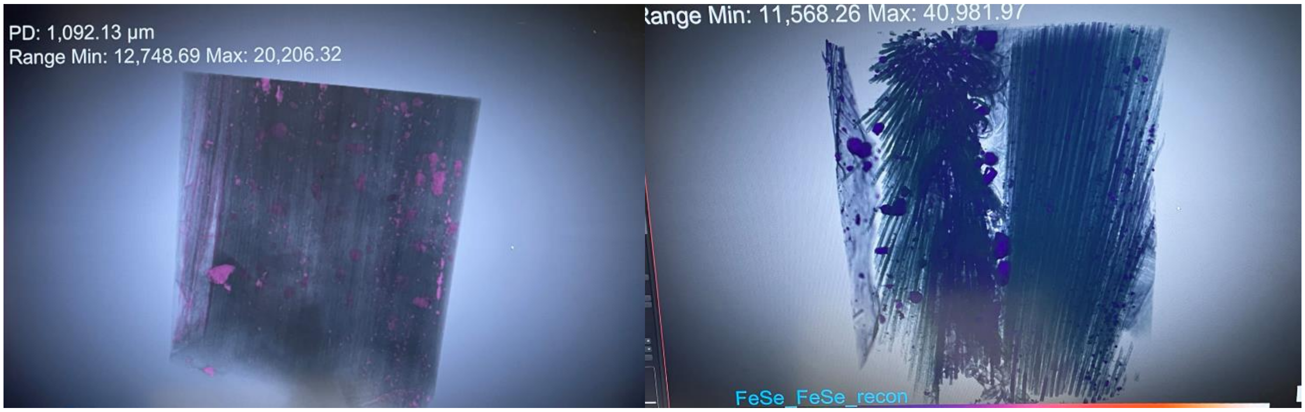

Figure S19. X-ray tomography of $FeSe_2$ inside the carbon fiber matrix before (left) and after (right) the cyclic test. $FeSe_2$ HPs are colored in each image.

Table S5. Comparison of cyclic stability test results of fabricated structural supercapacitor with the literature.

Structural Supercapacitor	No. of cycles tested	Capacitance retention	Coulombic efficiency	Capacitance at cycle 1	Method utilized	Reference
**CF/$FeSe_2$ HPs/Celgard/Epoxy**	**10,000**	**36.4% (at 10,000 cycles)**	**97.3%**	**77.6 $mF.g^{-1}$**	**GCD**	**This work**
CF/GF/PEGDGE	1000	85%	--	10.3 $mF.cm^{-3}$	Chronoamperometry	Javaid (2016)[40]
CF(PAN)/Celgard/1M $LiPF_6$ (liquid electrolyte)/Li	50	35.9%	--	3.2* $F.g^{-1}$	Capacity test at constant current	Snyder (2009)[41]
CF/FP/LiCl/PVA (gel electrolyte)	200	85%	--	32 $mF.cm^{-1}$	GCD	Qin (2017)[42]
CF/ZnO/GF/IL/LiTFSI (liquid electrolyte)	2500	93.2%	--	0.21* $F.g^{-1}$	GCD	Deka (2017)[43]
CF/NiCo/Teflon/ PEO-b-P(S-co-DVB) + BMIMTFSI	2000	90.2%	--	5 $mF.cm^{-2}$	GCD	Bae (2018)[44]
CF/GNP/MnO_2/ PVDF-HFP/$BMIMBF_4$/epoxy	2000	80%	--	15* $F.g^{-1}$	GCD	Masouras (2019)[45]
Graphene/Magnesium phosphate cement/KOH	2000	89.6%	--	40.9* $F.g^{-1}$	CV	Ma (2019)[46]
CF/CuCoSe/KF/IL/LiTFSI (liquid electrolyte)	2000	96.5%	--	28.6* $F.g^{-1}$	GCD	Deka (2019)[47]
CF/ $NiCo_2O_4$/PVA/KOH (gel electrolyte)	5000	72.5%	--	61.6* $F.g^{-1}$	GCD	Chen (2020)[48]
rGO/ANF/CNT/PP/6M KOH (liquid electrolyte)	5000	19.4%	--	169* $F.g^{-1}$	GCD	Patel (2020)[49]
CF/CuMnSe/KF/IL/LiTFSI (liquid electrolyte)	3500	94.4%	99%	47.3* $F.g^{-1}$	GCD	Deka (2020)[50]
CF/N_$ZnCuSe_2$-Mxene/ KF/IL/LiTFSI (liquid electrolyte)	4000	82.4%	79%	13.9* $F.g^{-1}$	GCD	Deka (2021)[51]
CF/SnS_2/CNT/PANI/1M Na_2SO_4 (liquid electrolyte)	6000	83.8%	--	891* $F.g^{-1}$	GCD	Zhang (2021)[52]
CF/$NiCo_2O_4$/KF/3M KCL (liquid electrolyte)	5500	--	94%	37.4* $F.g^{-1}$	GCD	Roh (2021)[32]
CF/CAG/GF/Epoxy/IL	900	90%	97%	2.17* $F.g^{-1}$	GCD	Qi (2021)[53]
CF/GF/Epoxy/PVDF/LiTFSI	6000	96%	--	0.13 $mF.cm^{-2}$	GCD	Xu (2021)[54]
CF/GF/Epoxy/IL	1000	90%	---	1.56* $F..g^{-1}$	GCD	Ding (2022)[55]
CF/GNP/Epoxy/$EMIBF_4$	1500	77%	--	0.62* $F.g^{-1}$	CV	Hubert (2022)[56]

CF/MnO_2/Silane/GF/IL (liquid electrolyte)	5000	92%	--	49 $mF.g^{-1}$	CV	Huang (2022)[57]
CF/CAG/GF/IL (liquid electrolyte)	3000	85%	97%	21 $F.g^{-1}$	GCD	Asfaw (2023)[58]

*Based on active mass of electrodes.

7. Supplementary References

[1] A. Javaid, S. Noreen, *J. Energy Storage* **2022**, *55*, 105818.

[2] Y. Cao, J. J. J. Chen, M. A. Barteau, *J. Energy Chem.* **2020**, *50*, 115.

[3] G. Kresse, J. Furthmüller, *Phys. Rev. B - Condens. Matter Mater. Phys.* **1996**, *54*, 11169.

[4] P. E. Blöchl, *Phys. Rev. B* **1994**, *50*, 17953.

[5] D. Joubert, *Phys. Rev. B - Condens. Matter Mater. Phys.* **1999**, *59*, 1758.

[6] A. Togo, I. Tanaka, *Scr. Mater.* **2015**, *108*, 1.

[7] A. Togo, L. Chaput, I. Tanaka, *Phys. Rev. B* **2015**, *91*, 94306.

[8] J. Shang, X. Gao, *Chem. Soc. Rev* **2014**, *43*, 7267.

[9] W. Haiss, N. T. K. Thanh, J. Aveyard, D. G. Fernig, *Anal. Chem.* **2007**, *79*, 4215.

[10] N. G. Khlebtsov, *Anal. Chem.* **2008**, *80*, 6620.

[11] D. Paramelle, A. Sadovoy, S. Gorelik, P. Free, J. Hobley, D. G. Fernig, *Analyst* **2014**, *139*, 4855.

[12] W. W. Yu, L. Qu, W. Guo, X. Peng, *Chem. Mater.* **2003**, *15*, 2854.

[13] *Electrochemical Impedance Spectroscopy (EIS) Part 1 – Basic Principles*, **2011**.

[14] *Electrochemical Impedance Spectroscopy (EIS) Part 2 - Experimental Setup*, **2011**.

[15] *Electrochemical Impedance Spectroscopy (EIS) Part 3 - Data Analysis*, **2011**.

[16] "FeSe2 Crystal Structure," **2020**.

[17] S. K. Haram, B. M. Quinn, A. J. Bard, *J. Am. Chem. Soc.* **2001**, *123*, 8860.

[18] W. J. Albery, P. N. Bartlett, J. D. Porter, *J. Electrochem. Soc.* **1984**, *131*, 2896.

[19] A.J. Bard, L. R. Faulkner, John Wiley & Sons, Inc., New York, **2001**.

[20] H. Fan, H. Yu, Y. Zhang, J. Guo, Z. Wang, H. Wang, N. Zhao, Y. Zheng, C. Du, Z. Dai, Q. Yan, J. Xu, *Energy*

Storage Mater. **2018**, *10*, 48.

[21] W. Zhao, C. Guo, C. M. Li, *J. Mater. Chem. A* **2017**, *5*, 19195.

[22] J. Xu, K. Jang, J. Lee, H. J. Kim, J. Jeong, J. G. Park, S. U. Son, *Cryst. Growth Des.* **2011**, *11*, 2707.

[23] N. S. Hudak, A. D. Schlichting, K. Eisenbeiser, *J. Electrochem. Soc.* **2017**, *164*, A691.

[24] A. Javaid, K. K. C. Ho, A. Bismarck, J. H. G. Steinke, M. S. P. Shaffer, E. S. Greenhalgh, *J. Compos. Mater.* **2018**, *52*, 3085.

[25] A. Javaid, M. Z. Ali, *Mater. Res. Express* **2018**, *5*, DOI 10.1088/2053-1591/AABEB1.

[26] A. Javaid, M. B. Zafrullah, F. ul H. Khan, G. M. Bhatti, *J. Compos. Mater.* **2019**, *53*, 1401.

[27] A. Javaid, M. Irfan, *Mater. Res. Express* **2019**, *6*, 016310.

[28] R. Reece, C. Lekakou, P. A. Smith, *Mater. Sci. Technol. (United Kingdom)* **2019**, *35*, 368.

[29] A. Ganguly, A. Karakassides, J. Benson, S. Hussain, P. Papakonstantinou, *ACS Appl. Energy Mater.* **2020**, *3*, 4245.

[30] A. Javaid, O. Khalid, A. Shakeel, S. Noreen, *J. Energy Storage* **2021**, *33*, DOI 10.1016/j.est.2020.102168.

[31] X. F. Sánchez-Romate, A. Del Bosque, J. Artigas-Arnaudas, B. K. Muñoz, M. Sánchez, A. Ureña, *Electrochim. Acta* **2021**, *370*, 137746.

[32] H. D. Roh, B. K. Deka, H. W. Park, Y. Bin Park, *Compos. Sci. Technol.* **2021**, *213*, 108833.

[33] K. Subhani, X. Jin, P. J. Mahon, A. Kin Tak Lau, N. V. Salim, *Compos. Commun.* **2021**, *24*, 100663.

[34] C. Fang, D. Zhang, *Chem. Eng. J.* **2021**, *426*, 130793.

[35] M. F. Pernice, G. Qi, E. Senokos, D. B. Anthony, S. Nguyen, M. Valkova, E. S. Greenhalgh, M. S. P. Shaffer, A. R. J. Kucernak, *Multifunct. Mater.* **2022**, *5*, 025002.

[36] E. S. Greenhalgh, J. Ankersen, L. E. Asp, A. Bismarck, Q. Fontana, M. Houlle, G. Kalinka, A. Kucernak, M. Mistry, S. Nguyen, H. Qian, M. Shaffer, N. Shirshova, J. Steinke, M. Wienrich, in *J. Compos. Mater.*, SAGE PublicationsSage UK: London, England, **2015**, pp. 1823–1834.

[37] A. Javaid, K. K. C. Ho, A. Bismarck, M. S. P. Shaffer, J. H. G. Steinke, E. S. Greenhalgh, *J. Compos. Mater.* **2014**, *48*, 1409.

[38] A. Javaid, K. K. C. Ho, A. Bismarck, J. H. G. Steinke, M. S. P. Shaffer, E. S. Greenhalgh, *J. Compos. Mater.* **2016**, *50*, 2155.

[39] E. S. Greenhalgh, S. Nguyen, M. Valkova, N. Shirshova, M. S. P. Shaffer, A. R. J. Kucernak, *Compos. Sci. Technol.* **2023**, *235*, 109968.

[40] A. Javaid, K. K. C. Ho, A. Bismarck, J. H. G. Steinke, M. S. P. Shaffer, E. S. Greenhalgh, *J. Compos. Mater.* **2016**, *50*, 2155.

[41] J. F. Snyder, E. L. Wong, C. W. Hubbard, *J. Electrochem. Soc.* **2009**, *156*, A215.

[42] T. Qin, S. Peng, J. Hao, Y. Wen, Z. Wang, X. Wang, D. He, J. Zhang, J. Hou, G. Cao, *Adv. Energy Mater.* **2017**, *7*, 1700409.

[43] B. K. Deka, A. Hazarika, Ob. Kwon, D. Y. Kim, Y. Bin Park, H. W. Park, *Chem. Eng. J.* **2017**, *325*, 672.

[44] S. H. Bae, C. Jeon, S. Oh, C. G. Kim, M. Seo, I. K. Oh, *Carbon N. Y.* **2018**, *139*, 10.

[45] A. Masouras, D. Giannopoulos, B. Hasa, A. Katsaounis, V. Kostopoulos, *J. Energy Storage* **2019**, *23*, 515.

[46] W. Ma, D. Zhang, *https://doi.org/10.1177/0021998318790322* **2018**, *53*, 719.

[47] B. K. Deka, A. Hazarika, J. Kim, N. Kim, H. E. Jeong, Y. Bin Park, H. W. Park, *Chem. Eng. J.* **2019**, *355*, 551.

[48] J. Chen, T. Ma, M. Chen, Z. Peng, Z. Feng, C. Pan, H. Zou, W. Yang, S. Chen, *J. Energy Storage* **2020**, *32*, 101895.

[49] A. Patel, D. Loufakis, P. Flouda, I. George, C. Shelton, J. Harris, S. Oka, J. L. Lutkenhaus, *ACS Appl. Energy Mater.* **2020**, *3*, 11763.

[50] B. K. Deka, A. Hazarika, S. Lee, D. Y. Kim, Y. Bin Park, H. W. Park, *Nano Energy* **2020**, *73*, 104754.

[51] B. K. Deka, A. Hazarika, M. J. Kwak, D. C. Kim, A. P. Jaiswal, H. G. Lee, J. Seo, C. Jeong, J. H. Jang, Y. Bin Park, H. W. Park, *Energy Storage Mater.* **2021**, *43*, 402.

[52] Z. Zhang, L. Feng, P. Jing, X. Hou, G. Suo, X. Ye, L. Zhang, Y. Yang, C. Zhai, *J. Colloid Interface Sci.* **2021**, *588*,

84.

[53] G. Qi, S. Nguyen, D. B. Anthony, A. R. J. Kucernak, M. S. P. Shaffer, E. S. Greenhalgh, *Multifunct. Mater.* **2021**, *4*, 034001.

[54] Y. Xu, S. Pei, Y. Yan, L. Wang, G. Xu, S. Yarlagadda, T. W. Chou, *ACS Appl. Mater. Interfaces* **2021**, *13*, 11774.

[55] Y. Ding, G. Qi, Q. Cui, J. Yang, B. Zhang, S. Du, *Energy and Fuels* **2022**, *36*, 2171.

[56] O. Hubert, N. Todorovic, A. Bismarck, *Compos. Sci. Technol.* **2022**, *217*, 109126.

[57] F. Huang, Y. Zhou, Z. Sha, S. Peng, W. Chang, X. Cheng, J. Zhang, S. A. Brown, Z. Han, C. H. Wang, *ACS Appl. Mater. Interfaces* **2022**, *14*, 30857.

[58] H. D. Asfaw, A. Kucernak, E. S. Greenhalgh, M. S. P. Shaffer, *Compos. Sci. Technol.* **2023**, *238*, 110042.
